# Uncovering the Molecular Machinery of the Human Spindle—An Integration of Wet and Dry Systems Biology

**DOI:** 10.1371/journal.pone.0031813

**Published:** 2012-03-09

**Authors:** Ana M. Rojas, Anna Santamaria, Rainer Malik, Thomas Skøt Jensen, Roman Körner, Ian Morilla, David de Juan, Martin Krallinger, Daniel Aaen Hansen, Robert Hoffmann, Jonathan Lees, Adam Reid, Corin Yeats, Anja Wehner, Sabine Elowe, Andrew B. Clegg, Søren Brunak, Erich A. Nigg, Christine Orengo, Alfonso Valencia, Juan A. G. Ranea

**Affiliations:** 1 Centro Nacional de Investigaciones Oncológicas, Structural Biology and Biocomputing Programme, Madrid, Spain; 2 Max Planck Institute for Biochemistry, Department of Cell Biology, Martinsried, Germany; 3 Center for Biological Sequence Analysis, Department of Systems Biology, Technical University of Denmark Kemitorvet, Lyngby, Denmark; 4 Department of Biochemistry and Molecular Biology, University of Malaga, Malaga, Spain; 5 Memorial Sloan-Kettering Cancer Center, Computational Biology Center, New York, New York, United States of America; 6 Department of Structural and Molecular Biology, University College London, London, United Kingdom; Virginia Tech, United States of America

## Abstract

The mitotic spindle is an essential molecular machine involved in cell division, whose composition has been studied extensively by detailed cellular biology, high-throughput proteomics, and RNA interference experiments. However, because of its dynamic organization and complex regulation it is difficult to obtain a complete description of its molecular composition. We have implemented an integrated computational approach to characterize novel human spindle components and have analysed in detail the individual candidates predicted to be spindle proteins, as well as the network of predicted relations connecting known and putative spindle proteins. The subsequent experimental validation of a number of predicted novel proteins confirmed not only their association with the spindle apparatus but also their role in mitosis. We found that 75% of our tested proteins are localizing to the spindle apparatus compared to a success rate of 35% when expert knowledge alone was used. We compare our results to the previously published MitoCheck study and see that our approach does validate some findings by this consortium. Further, we predict so-called “hidden spindle hub”, proteins whose network of interactions is still poorly characterised by experimental means and which are thought to influence the functionality of the mitotic spindle on a large scale. Our analyses suggest that we are still far from knowing the complete repertoire of functionally important components of the human spindle network. Combining integrated bio-computational approaches and single gene experimental follow-ups could be key to exploring the still hidden regions of the human spindle system.

## Introduction

Cell division is essential to life and understanding the molecular mechanisms controlling this process remains a major challenge. From the perspective of cell structure and dynamics the separation of the chromosomes during mitosis, and the process of cell division (cytokinesis), represent dramatic events in the lifespan of the cell. Both chromosome separation and cytokinesis are dependent on a highly dynamic microtubule based structure, the mitotic spindle [1].

The spindle apparatus presents a challenging problem for Systems Biology, as its formation involves many different structural and regulatory molecules. Spindle-associated proteins cover a broad range of functional categories as they can be mechanical and structural components; cargo proteins transported by the spindle apparatus; as well as proteins involved in the regulation of spindle assembly.

Capturing this complexity poses a great challenge for any type of experimental or bioinformatics approach. Specific experimental approaches together with large scale proteomics have contributed substantially to the characterization of the spindle components [2]. Furthermore, a number of large-scale siRNA experiments in various model systems have detected potential regulators of spindle morphology and cell cycle progression [3], [4].

Whilst major progress has been made in deciphering the temporal and spatial regulation of the mitotic spindle [5], [6], [7], [8], [9], [10], [11], [12], [13], [14], [15], it is uncertain whether the full repertoire of spindle, kinetochore and centrosomal proteins is known. Here, we have developed a combined bioinformatics and experimental strategy to identify some missing components of this important molecular system.

To identify novel spindle components and new protein functional association we developed a computational platform, called SPIP (Spindle Predictions Integrated Platform), integrating a variety of orthogonal methods ranging from neural networks to analysis of co-occurrences in publications (**Results section 1**). We benchmarked our approach both computationally using a statistical framework (**Results section 2**) and experimentally (**Results section 3**). We show that our approach accurately predicts novel spindle components and provides valuable additional material for characterising this system. Our results confirm the power of integration methodologies to predict the molecular players in biological systems. This has also been demonstrated in other biological scenarios [16], [17], [18], as well as in the MouseFunc competition [19], [20], [21], [22], where different groups set out to functionally annotate all currently uncharacterized mouse proteins and in recent studies of mitotic chromosome associated proteins in different organisms [15].

Furthermore, analysis of the network of interactions that we generate between previously known and new putative spindle proteins reveals the potential role of highly connected proteins that may play an essential role in the organization of the spindle machinery. Some of those highly connected proteins are still poorly characterised (hidden hubs), and this makes them particularly interesting. In summary, our combined experimental and computational analyses, together with the study of these ‘*hidden spindle nodes*’ suggests that a large number of novel and important components, needed for the organization of spindle system, remain to be fully characterized.

## Experiment

Methods used in this work can be classified into two types: one type for predicting functional associations between pairs of proteins and another type for predicting functions of individual proteins.

### Methods to predict functional associations between pairs of proteins

#### 1. The CO-CItations TExt mining method (COCITE)

PubMed is a comprehensive source of information about interactions described in the scientific literature [23]. The COCITE method identifies co-occurrences by using genes and proteins as hyperlinks between sentences and abstracts in PubMed [24]. Direct and indirect associations are calculated from these relationships and a score is calculated to rank the proteins. The rationale is to score proteins according to the number of times that they appear as interacting with each other. For this purpose we have extracted pairs of interactors from the whole human gene interaction network using iHOP [24]. We filtered the iHOP network by only considering those interactions that had a relationship described by verbs classified as “physical” in the sentence. This gave a total of 11,722 pairs of interacting proteins showing an interaction defined as “physical”.

Using the filtered network we performed two distinct calculations (see **Fig. S6**):

Direct interactions (S1 score or d-COCITE score).

The S1 score measures the strength of the direct associations between a predefined reference set of proteins and any given protein in order to establish whether it could be an unknown member of the set. The S1 score is intended to consider the specificity of the co-citations found and therefore it includes both the interactions of the protein with the reference set and those found with unrelated proteins. The rationale of this specificity-focused approach is to compensate the influence of those highly unspecific (“sticky”) proteins.

S1 is calculated for any reference set (*R*) and any protein (*i*) as follows:
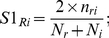
(1.1)where:

- *n_ri_* is the number of interactions between the protein *i* and proteins (*r*) of the reference set;
- *N_r_* is the total number of interactions of those proteins (*r*) in the reference set interacting with protein *i*;
- *N_i_* is the total number of interactions of protein *i*.

Consequently, the higher the score S1, the higher the number of specific interactions between the reference set and protein *i*. In this regard, we are accounting for all the possible protein-protein interactions in the human proteome.

Indirect interactions (S2 score or i-COCITE score).

In order, to improve both the sensitivity and specificity of our approach, we decided to include information about indirect interactions (distance = 2) between the reference set and the protein considered. These indirect interactions complement the direct ones because they provide information about the context of the network surrounding the corresponding protein. In this case we formulate the score S2, as an extension of the previously explained S1, considering those proteins c connecting the reference set to the protein i. Therefore S2 is calculated as follows:
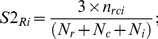
(1.2)where:

- *n_rci_* is the number of indirect interactions (distance = 2) between the protein *i* and proteins (*r*) of the reference data set;
- *N_r_* is the total number of interactions of those proteins (*r*) in the reference set interacting with proteins (*c*) that interact with protein *i*;
- *N_c_* is the total number of interactions of those proteins interacting with proteins of the reference set and with protein *i*;
- *N_i_* is the total number of interactions of protein *i*.

Consequently, the higher the score S2, the higher the number of specific indirect interactions between the reference set and protein i (method performance assessment is shown in **Text S1 section 9 and Figure S8**).

### 2. Co-occurrence Domain Analysis (CODA)

Co-Occurrence of Domains Analysis (CODA) uses a Multi-Domain Architecture [25] representation of proteins in complete genomes (target genomes) from Gene3D [26] to discover pairs of proteins involved in common biological processes within a complete genome of interest (the query genome). It is a novel approach in the domain fusion idiom using a new scoring method. The CODA method has been published, and its implementation and validation, as used in this work, is extensively described in Reid et al. 2010 [25].

The basic principle behind this method comes from the observation that some pairs of genes have become fused during evolution. The orthologues of distinct genes from one organism are sometimes found as a single gene in another organism. These genes tend to be functionally related, e.g. part of the same protein complex. Thus, functional inferences can be made between the protein pairs in an organism of interest based on fusion events observed in the genomes of other organisms.

As a more formal explanation of how CODA works we will consider how the method can predict a functional association for a particular pair of proteins *p*, *q* in a query genome *g*. *D_p_* is the set of domains in protein *p*. *a* 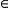 *D_p_* denotes that protein *p* contains a domain of superfamily *a*. *J_ p,q_* is the set of domain pairs *a, b* where *a* 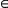 *D_p_*, *b* 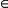 *D_p_*. In other words *J* consists of all the distinct pairs of domains between proteins *p* and *q*. So if D_p_ = {a, c} and D_q_ = {b}, J_p,q_ = {ab, cb}. It is also required that *a≠b* as the two proteins must not share any domains of the same superfamily. When two proteins share domains from the same superfamily they are ignored.

To determine a fusion event we require that a target genome *t* (one other than the query genome) contains a protein *r* with domains *a* and *b* such that *ab* 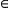 *J_p,q_* i.e. domains which are separated in the query genome are found fused in the target genome. The set *T* comprises those genomes other than *g* which contain such proteins *r*. For a domain pair *a,b* in genome *g*, the fusion score *s_a,b_* is taken as a maximum over all genomes in *T*:
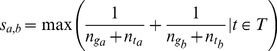
(3.1)where *|T|* is the number of elements in set *T* (i.e. the number of target genomes), 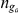 and 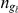 are the frequencies of domain *a* and domain *b* respectively in genome *g* and 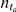 and 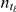 are the frequencies of domains *a* and *b* respectively in genome *t*. The score *s_a,b_* is not dependent on a particular pair of proteins, but only a particular pair of domain families in genome *g*. For a particular protein pair *p,q*, in query genome *g*, the maximum *s_a,b_* is taken over all domain pairs *a,b* in *J_p,q_*.
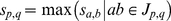
(3.2)


Thus *s_p,q_* is the CODA score for proteins *p*,*q*; the best (highest) score over all domain pairs between the proteins and over potential fusion proteins in all genomes (other than the query genome). Validation of CODA score performance in predicting protein-protein functional associations has previously been performed using the yeast proteome annotated in GO [25].

#### 3. hiPPI (homology inherited Protein-Protein Interaction)

The hiPPI method uses a homolgy based approach to inherit interactions between pairs of proteins. The Gene3D resource contains protein families sub-clustered at 11 different levels of sequence identity using multi-linkage clustering (0, 10%, 20% up to 100% seq. id. - the clusters are termed the “S-levels” and numbered 1–11) (described in detail in Ranea et al., 2010 [27]). Known interactions, along with the experimental evidence type, gathered from MIPS, IntAct, HPRD and MINT, have been included for each protein family [26].

In the first step all interactions are transferred (“inherited”) to homologues in the same pair of protein families (“A” and “B”), implying that any member of family A could interact with any member of family B. Then each potential interaction is evaluated with a simple scoring method that takes into account the evolutionary distance of the predicted pair from the proteins involved in the experimentally determined (known) interactions, the number of supporting experimental types and number of species the interaction is seen in.

The evolutionary distance is measured by identifying the sequence identity cluster (eg. S-Level 10, sequence identity 90%) to which the inheriting protein and the protein with the known interaction data, belong, for each partner in the pair. The score is then obtained by averaging the numbers associated with the particular S-levels. For example when inheriting interactions from the pair A_1_-B_1_ to the pair A_2_-B_2_, and if the protein A_2_ is within a 100% sequence identity cluster with A_1_ (S-level 9) and the protein B_2_ is within a 80% cluster with B_1_ (S-level 7) then the score will be (9+7)/2 = 8. This ensures that interactions inherited from distant homologues, at a low S-level, contribute less than those inherited from close homologues.

The score is further increased if the pair of proteins with known interactions have multiple interaction data, ie. the interaction is supported by different experimental types or found in a different species. In this case for each extra species or extra experiment type a score half as much as previous is added. For example, if the score for a predicted pair is 8, then if the known interaction data comes from two independent sources then an extra score of 4 is added, if the interaction is in more than one species then a further 2 is added and so on. Thus the fact that interactions may be experimental false positives unless well supported, or only occur in a single species and not in others, is also reflected in the final score.

The final score for a predicted interaction is the sum of the scores for all the supporting interactions (ie for predicted pair A_1_-B_1_, supporting data from known interactions could come from pair A_2_-B_2_ and also from pair A_3_-B_3_ whose partners are in different clusters to proteins in A_2_-B_2_).

Full details of the hiPPI method and its implementation and validation, as used in this work, are described in Ranea et al. 2010 [27].

#### 4. Gene Expression COrrelation (GECO) method

Microarrays provide a high throughput approach for identifying functionally related proteins. We have made use of GECO, which simply measures the Pearson correlation coefficient of gene expression profiles between known and putative spindle proteins. For human we use the E-TABM-185 compendium dataset of ∼6000 GCRMA normalised HGU133-A Affymetrix microarrays assembled by array- express [28]. A maximum of 5 values were allowed to be missing from a given genes expression profile, using the C-clustering libraries masking function. For the human HGU133a Affymetrix chips 14,500 genes are well characterised giving a very large set of similarity scores. Further details of the methods implementation and validation as is described in Ranea et al. 2010 [27].

#### 5. Gene Ontology Semantic Similarity (GOSS) method for validation

To validate the SPIP method, we chose to analyse our predictions with the Gene Ontology (GO) database, which allowed us to implement a consistent measure of the functional relationships between known spindle and the other proteins in the human proteome. A Gene Ontology semantic similarity (GOSS) score was calculated for each protein pair using an implementation of the Resnik method described in [27], [29]. This implementation and validation of this methods, as used in this work is described in Ranea et al., 2007 and Ranea et al., 2010 [27], [29].

### Methods to predict functions of individual proteins

#### 6. Data driven machine-learning based on artificial neural networks (MLNN)

For training the first version of the mitotic spindle predictor a set of proteins identified as either spindle proteins or likely contaminants, 151 and 517 respectively, were compiled [2]. The data set was homology reduced, using an approach developed elsewhere [30], [31] yielding a final data set of 341 proteins with a 2∶9 ratio of positive to negative examples. In brief, the homology reduction removes proteins with a protein sequence too similar to the other proteins in the data set. The data set was used in three-fold cross validated training of a feed forward neural network. For each protein in the data set, 43 protein features were predicted and calculated by a variety of computational tools. The protein features include amino acid content, post-translational modifications (such as S/T phosphorylation, kinase-specific phosphorylations, and N-linked glycosylation), subcellular localization, signal peptides, degradation signals, physio-chemical properties (such as Isoelectric point) and presence and number of transmembrane helices. From this set of features, those with discriminatory power with respect to the spindle classification were identified by using each feature alone as input to the neural network and subsequently recording the Matthews correlation coefficient (a two-class discretized version of the Pearson correlation coefficient) on the test part of the data set. Features performing well were combined in pairs and used as input to the neural network and their combined performance evaluated. Triplets were generated from the well performing feature pairs, until no additional performance was gained. In total, 12 artificial neural networks using four different feature combinations were constructed and the predictions from these neural networks were combined in an ensemble, which make up the first version of the spindle predictor. This ensemble of predictors was applied to the entire human proteome as well as the set of proteins purified with the mitotic spindle by Sauer *et al.* [2] to identify novel, potential spindle proteins.

A second version of the predictor was trained on an updated data set of proteins, where novel spindle proteins, including some from the validation experiments described in section four, were included. After manual curation of the data sets it consisted of 467 negative and 146 positive examples. The data set was homology reduced as described above yielding a final data set of 305 proteins with a one-to-four ratio of positive to negative examples. This updated data set was used to train a novel, updated version of the prediction method including additional features, such as a coil-coil structural feature [32], [33]. Based on the new data set and the extended pool of protein features, the method was retrained as described above. The best performing combinations of features were used as input to 4 different networks as depicted in **Fig. S7**.

To obtain the best predictive performance the two prediction methods were combined into a final spindle predictor. This prediction method is available through a web server (http://www.cbs.dtu.dk/services/SpindleP). To test the performance of the combined predictor, an evaluation data set was generated as follows. As positive examples, 100 proteins generating a mitotic phenotype upon knock out were selected under the assumption that this set of proteins will be enriched for spindle proteins. As a negative data set 529 random proteins were selected. The data set was homology reduced such that no strong homology exists among proteins within each category. Performance of both the individual predictors and the combined predictor was evaluated using the area under ROC curve as performance measure on the evaluation set. A comparison of the performance revealed that the area under ROC curve increased by 0.05 for the combined prediction method compared the individual versions.

#### 7. Domain Over-Representation Analysis (DORA)

This method searches for specific spindle domains in the target proteins using Pfam domain annotations from the Gene3D database [26]. DORA score (Cij) measures the ratio of the relative frequency of a given domain *i* of protein *j* in the spindle set (see Ft/Nt in formulae 2.1) compared to the relative frequency of the same domain in the whole human proteome (Fb/Nb).
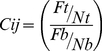
(2.1)

- Cij is the score for a particular family (Pfam domain superfamily) *i* presents in the protein *j*.
- Ft is the frequency of that family *i* in the target group of proteins e.g. the spindle proteins data set SEED.
- Nt is the total number of domains in the target group.
- Fb is the frequency of that family *i* in all proteins in the genome.
- Nb is the total number of domains in the genome.

Therefore high numbers suggest that a domain is associated with the target data set (e.g. to spindle function) and therefore that novel proteins containing that domain are more likely to be involved in the spindle.

## Results

### 1. Building an integrated platform for predicting human spindle proteins

**Figure 1:**
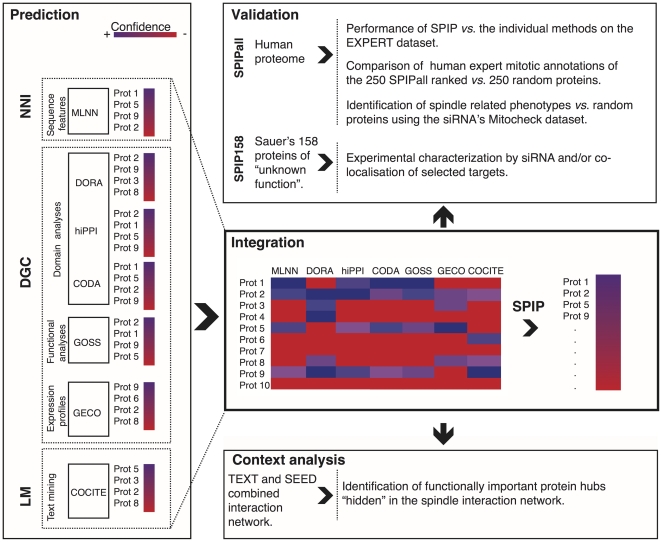
The Spindle Prediction Integrated Platform (SPIP) workflow. Left panel, “Prediction”: describes three different approaches (dashed boxes, NNI, DGC, LM) which include seven independent methods for predicting spindle associated proteins from all proteins in the human proteome. Each method has its own associated confidence score (red: the less confident). NNI group of methods includes the MLNN method that integrates different spindle protein features to predict new spindle proteins using Neural Network technology; The DGC approach includes the following methods: DORA that searches for domains characteristic of known spindle proteins in target proteins; hiPPI that scores potential interactions between putative and spindle proteins based on their homology to known interacting protein pairs; CODA that scores putative spindle proteins if there is a homologous domain fused to a homologue of a domain typically associated with spindle proteins; the GOSS method that measures semantic similarity of the GO terms for known and putative spindle proteins, and finally the GECO method that measures the correlation of gene expression profiles between known and putative spindle proteins. The LM approach includes the COCITE method that detects pairs of spindle and target proteins co-cited in the literature. The left panel of the figure represents the following: For a given set of proteins (labelled with numbers) each method scores the same protein at a different rank, for example protein 1 is top-ranked in NNI but ranked in second place by Hippi i.e. depending on the method we could have different rankings for the same protein. Central box, “Integration”: The scores within each prediction dataset were translated into p-values and combined in a target prediction matrix. The prediction p-values from the 3 approaches, LM, NNI and DGC were then integrated into the Spindle Prediction Integrated Platform score (SPIP) for every protein target, again using Fisher's method (for more details see the Material and Methods section). Upper box, Validation”: SPIP was validated using two different schemes, a computational one using the whole human proteome, and an experimental one using a subset of selected “unknown proteins” to conduct experimental validation (see the text). Lower box, “Context analyses”: to identify relevant targets potentially involved in “hidden hubs”.

The seven different computational methods, described above, were integrated to predict potential spindle proteins (**Fig. 1**). The methods base their predictions on very different types of information, and can be grouped into three main categories: literature mining methods (**LM** methods - COCITE); neural network inference methods (**NNI** methods - MLNN) and domain and genomic context methods (**DGC** methods – comprising CODA, DORA which use protein domain annotations at various levels, GECO which is based on the analysis of gene expression, hiPPI which infers protein interactions from the analysis of homology relations, and GOSS which is based on the analysis of protein semantic similarity in the GO database, see **Fig. 1**). Although CODA and DORA base their predictions on protein domain annotation, the evolutionary and functional signals they exploit and the nature of their prediction outcomes are different. CODA searches for domain fusion events which have occurred in the evolution of some species and yields protein pair association predictions; while DORA looks for spindle functional domains in the target set yielding predictions for single protein targets.

The methods were seeded with a set of 149 well-characterized human spindle proteins (the SEED dataset) obtained by manual curation of proteins in the Sauer proteomics dataset [2], i.e. proteins that had already been reported as being spindle associated, in the literature (**Methods section 1**). The application of the seven methods to various databases and conditions produced eight sets of predictions (one of the methods, i.e CODA produced two data sets based on CATH and PFAM domain annotations respectively, see **Methods section 2** for details). The results were integrated by the Spindle Predictions Integrated Platform (SPIP) into a single prediction list with a unified *p*-value calculated using the classical Fisher's meta-statistics method (**Fig. 1**, see also **Methods section 2**). The integrated predictor takes into account the heterogeneity of the methods' formats and scores. One of the reasons to select Fisher statistics amongst other choices (e.g. Bayesian methods, such as, for example, the Naïve-Bayes classifier that has to be trained in a supervised learning setting) was that Fisher's integration does not require training on experimental data unlike the Naïve-Bayes classifier algorithm. This feature avoids or reduces the dependency on the experimental data. We considered this property of Fisher's method a desirable feature for detecting novel spindle components and for benchmarking the performance of our approach.

The final list of predicted spindle proteins in the human proteome (**SPIPall dataset**) contains scores (p-values, **Table S1**) for 32,145 proteins. The pre-computed spindle predictions from all the methods and from the integrated predictor are available for public use in the form of a web server (**Text S1 section 4**).

### 2. Statistical assessment of the performance of the integrated prediction platform applied to the human proteome (SPIPall predictions)

Three different benchmarks were performed using statistical frameworks and validating against sets of proteins known to be spindle associated:

#### i. Benchmarking SPIP using the EXPERT dataset of curated spindle proteins

The performance of the three basic approaches (literature-mining **LM** (CO-CITE); domain and genomic context comparison **DGC** (GOSS, CODA, DORA, GECO, hiPPI); and neural network inference **NNI** (MLNN) (**Table 1** **and** **Fig. 1**)) and the integrated platform (SPIP) were validated using an independent data set of spindle proteins well supported in the literature (EXPERT, see **Methods section 1**). EXPERT is a manually curated set of spindle proteins not present in the SEED dataset (**Methods section 1**) [2] used to train the methods.

**Table 1:** Summary of the methods used in this study.

Class	Method	Type	Laboratory
Literature Mining (LM)	CO-CITE (direct & indirect)	Prediction	CNIO
Neural Networks Inference (NNI)	MLNN	Prediction	DTU
Domain and Genomic Context (DGC)	GOSSDORACODA(pfam & cath)hiPPIGECO	Prediction	UCL
COMBINED	SPIP	PredictionIntegration	UCL
TEXT	Literature mining-SVM	Validation	CNIO
EXPERT	Literature mining-SVM+Manual evaluation	Validation	CNIO

The class, method, type and laboratory where the methods were developed is shown.

**Figure 2:**
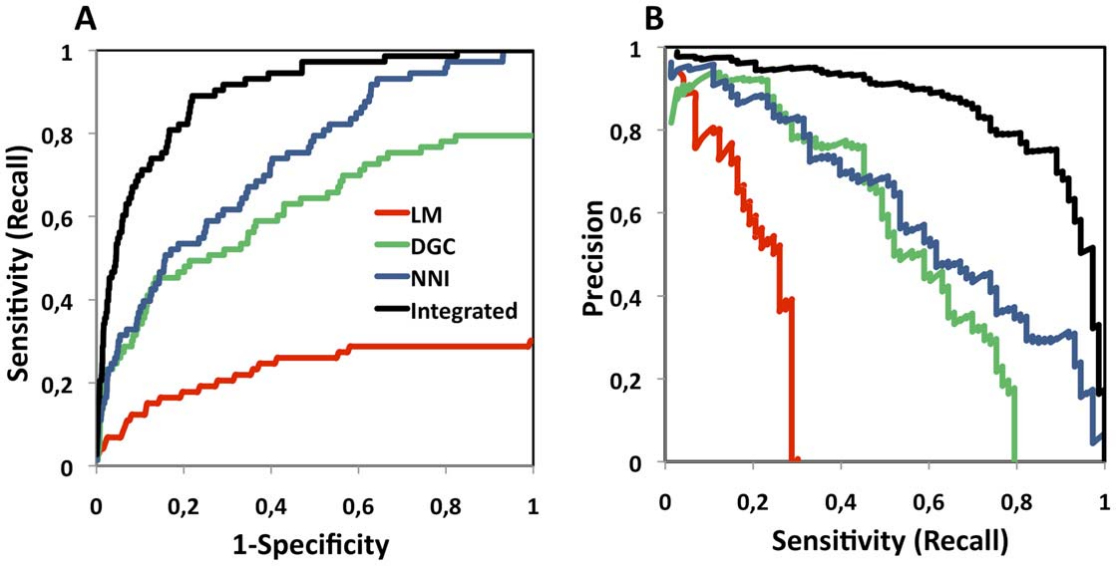
Validation of the performance of the spindle prediction platform (SPIP) in the human proteome. Validation of the predictions using the text mined, manually curated dataset, EXPERT, as true positives. (A) ROC curves: Sensitivity (also called Recall; y-axis) versus 1-Specificity (x-axis). And (B) PR curves: Precision (y-axis) versus Recall (x-axis) retrieved by each method.

The integrated method SPIP significantly outperformed the sensitivity (recall), specificity and precision of the independent methods (Fig. 2) and all of their pair-wise combinations (Fig. S1) predicting true spindle function, as can be seen in the Receiver Operator Characteristic (ROC) curves, (Fig. 2A; Fig. S1A and Table S2) and in the Precision-Recall (PR) curves (Fig. 2B, Fig. S1B and Table S2). All the integrated methods, including the three high-level approaches: LM, NNI and DGC, yielded highly independent predictions when compared to each other. The independence of the datasets was checked by performing a statistical analysis of mutual information (Tables S3 and S4). The independence of the datasets is an important requirement for ensuring that the Fisher integration score does not overestimate the statistical significance of the predictions. Therefore, the higher performance given by Fisher's integration indicates the efficiency of this method in combining the complementary information that the different methods provide.

#### ii. Benchmarking SPIP by analysing the enrichment of spindle proteins in the ranked list

Since experimental validation of the complete list of thirty two thousand predicted proteins was not feasible, manual validation was performed by human analysis of the 500 top ranked proteins. We observed a 1.6 times increase in mitotic-related proteins in the first 50 ranked proteins compared to the subsequent 50. Repeating this strategy with the top 100 and subsequent 100, we achieve an enrichment of 1.76. The same is true when we compared the 250 top ranked proteins to the subsequent 250. Here we observed an enrichment of 2.6. From these observations we can deduce that the ranking strategy works and that a lower rank for any given protein signifies a higher chance of being a spindle protein.

Our enrichment analysis is strict since it only considers known spindle proteins and assumes all the others are ‘false positives’, even if they could be currently uncharacterised spindle proteins. In addition, we observed that the number of mitotic-related proteins identified in the top 250 ranked list by SPIP (68 proteins; **Table S5**) is significantly higher when compared with the numbers identified in two random sets of 250 proteins (1 and 3 proteins, respectively).

### iii. Benchmarking SPIP by analysing the enrichment of Mitocheck phenotypes in the ranked list

Additional benchmarking was performed using the Mitocheck siRNA experiments and related phenotype data [34]. MitoCheck used RNA interference (RNAi) high-throughput screens to identify all proteins required for mitosis in human cells. Since Mitocheck also contains phenotype categories which are not specific to spindle genes, such as “Cell death” or “Large”, we mapped the human spindle ranked list with a subset of Mitocheck phenotype categories more closely related to spindle gene malfunctions in the cell cycle, such as: “Segregation problems”, “Metaphase alignment problems” and “Metaphase delay/arrest”. In total 361 mitocheck genes with any of these phenotypes were mapped onto the 32,145 human proteome spindle ranked list.

Enrichment was calculated by dividing the number of TPs by the number of FPs found at the same rank threshold (see **Fig. S2**). Runtest and Random test indicated a highly significant enrichment at the top of the rank list, with about 10 fold enrichment of the Mitocheck spindle associated phenotypes (see **Text S1 section 5, Fig. S2, Fig. S9 and Table S11**). A result of all benchmarking experiments can be seen in **Table 2**.

**Table 2:** Summary of the benchmarks conducted in this study and the corresponding findings.

Scheme	Output	Benchmark	Validation	Results & Significance test
SPIP run on whole Human Proteome	(SPIPall) protein list ranked by p-values	Performance of the integrated, single and combined prediction methods compared with random.	EXPERT dataset	ROC curves showing best performance for SPIP integrated SAUC statistics (**Figs. 2** and **S1**, and **Table S**2)
SPIP run on whole Human Proteome	(SPIPall) protein list ranked by p-values	Compare 250 top ranked proteins against second 250 ranked proteins and against random set.	Human annotation of mitotic-related proteins	2.6 fold increase in mitotic-related proteins in the first 250 compared to second 250.22-fold increase compared to random.(**Table S5**)
SPIP run on whole Human Proteome	(SPIPall) protein list ranked by p-values	Spindle-related phenotype (according to Mitocheck) enrichment versus random.	Mitocheck siRNA experiments	∼10 fold enrichment of Mitocheck phenotype proteins at the top of the SPIPall ranked list.Runtest and Random test statistics(**Figs. S2 and S3**)
SPIP run on the 158 unknown proteins (SAUER dataset)	The 158 proteins in the Sauer set ranked by SPIP (SPIP158)	Comparison of SPIP ranking for selecting targets with the procedure used in the Sauer et al analysis.	Experimental validation of the selected targets: SiRNAs and/or Co-localization with spindle	∼70% of success rate as compared to previous ∼35% success rate. **Fig. 3** and **Fig. S4**.

The scheme, output, benchmark, validation and results are shown.

### 3. Experimental validation of novel spindle proteins identified by the SPIP integrated platform

In addition to the benchmarking described above we also performed experimental validation of some of the proteins predicted by SPIP as the most likely candidates to be spindle associated. We selected these proteins from the 158 functionally unknown proteins previously identified by Sauer et al from a proteomics experiment [2]. This benchmark had the advantage of allowing us to compare the success rate of our predictions with that achieved by Sauer et al in selecting putative spindle associated proteins (see **Text S1 section 3**).

**Figure 3:**
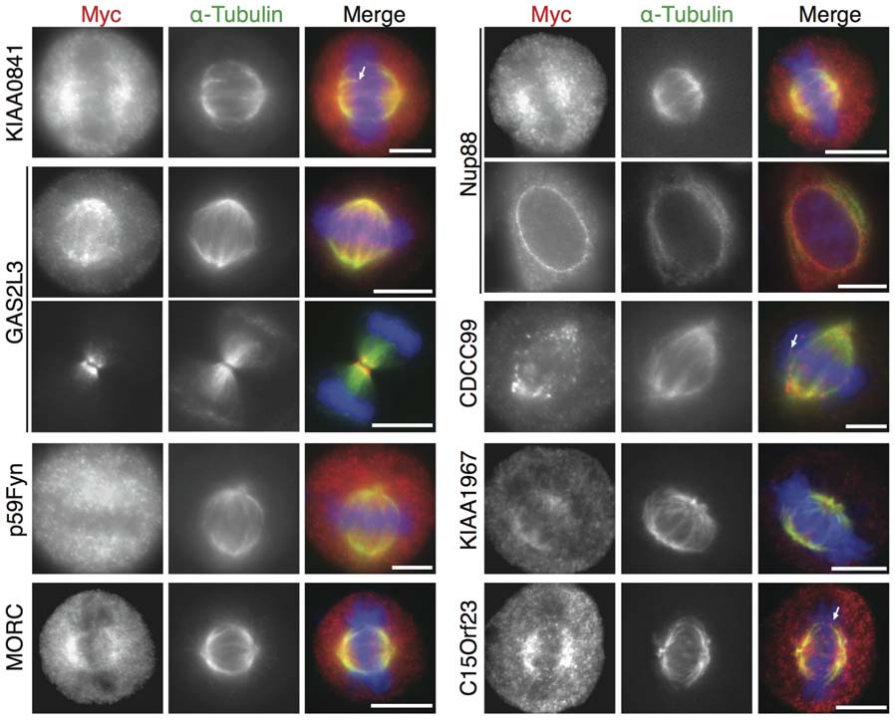
Mitotic localization of selected predicted candidate spindle proteins. HeLa S3 cells were transfected with the indicated myc-tagged constructs, fixed and analyzed by indirect immunofluorescence. Cells were stained with 9E10 anti-myc antibody (red) and with α-Tubulin (green). DNA was visualized using DAPI (blue). Arrows indicate positive kinetochore staining. Bar = 10 µm.

We selected 20 proteins from the top of the ranked list of SPIP predictions based on their amenability to experimental characterisation (see **Methods section 3** and **Table S6** for a detailed description). The ranks of the proteins selected can be seen in **Table 3**, the highest rank selected was rank 2 and the lowest selected was rank 62. 14 of the 20 proteins were successfully cloned, using the kinetochore protein *C1Orf48* gene (CA048_HUMAN [35] as positive control (**Fig. S4A**). We found 8 proteins (out of 14) localising to the spindle apparatus (kinetochores, spindle poles or microtubules; **Fig. 3**, **Table 3**), namely GA2L3, p59Fyn, Nup88, CDCC99, KIAA1967, C15orf23, MORC2, KIAA0841, in addition to the positive control. In contrast, 3 proteins (WDR76, WDR75 and Pescadillo homologue 1) showed chromosomal staining (see **Fig. S4B** and **Table 3**) and 2 proteins (SHCBP1, MK13) localized diffusely to the cytoplasm (**Fig. S4C** and **Table 3**).

**Table 3:** Targets selected for experimental validation and summary of mitotic localization and siRNA phenotypes.

Protein Name	Acc. No.	Cloned in this study	Mitotic localization (IF)	Localisation spindle apparatus (IF)	mitotic phenotype (siRNA)	SPIP rank
SHC SH2 domain-binding protein 1	SHCBP_HUMAN	yes	no spindle localization	No	No	24
WD repeat protein 76	WDR76_HUMAN	yes	Chromosomes	No	Yes	29
Pescadillo homologue 1	PESC_HUMAN	yes	Chromosomes	No	Yes	21
Mitogen-activated protein kinase 13	MK13_HUMAN	yes	no spindle localization	No	Yes	37
WD repeat protein 75	WDR75_HUMAN	yes	Chromosomes	No	Yes	31
GAS2-like protein 3	GA2L3_HUMAN	yes	spindle MTs and midbody	Yes	Yes (also [4])	33
Tyrosine kinase p59fyn	FYN_HUMAN	yes	spindle MTs	Yes	Yes	17
Nucleoporin 88	NUP88_HUMAN	yes	spindle MTs	Yes	Yes	2
Coiled-coil domain containing 99	CCD99_HUMAN	yes	spindle poles and kinetochores	Yes	Yes (also [45])	26
KIAA1967 (DBC1)	K1967_HUMAN	yes	spindle MTs	Yes	Yes (also [69])	27
C15orf23	T4AF1_HUMAN	yes	spindle MTs and kinetochores	Yes	Yes [46] (also [4])	50
MORC family CW-type zinc finger protein 2	MORC2_HUMAN	yes	spindle MTs (shown as MAP in [36]	Yes (also [36])	Yes	62
KIAA0841	K0841_HUMAN	yes	spindle MTs (and kinetochores weakly)	Yes (also [13])	Yes (also [4])	57
C1orf48 (positive control)	CA048_HUMAN	yes	kinetochores [35]	Yes [35]	Yes	16
Putative Nucleoporin protein 54	NUP54_HUMAN	No	Nd	Nd	nd	14
ZMYM1 protein	Q8N3X8_HUMAN	No	Nd	Nd	nd	41
KIAA1794	K1794_HUMAN	No	no spindle localization [36]	No [36], [44]	nd	51
Ser/thr-protein phosphatase 1 reg.sub. 10	PP1RA_HUMAN	No	nd (shown as MT-binding protein in [36]	Yes [36]	nd	35
C14orf106	CV106_HUMAN	No	kinetochores [45]	Yes [45]	Yes [45]	30
Echinoderm microtubule-associated protein-like 3	EMAL3_HUMAN	No	spindle MTs [36]	Yes [36]	Yes [36]	45

nd = not determined. The protein name, accession number, whether it has been successfully cloned in this study or not, the mitotic localization, whether they localize or not to the spindle apparatus, the reported siRNA phenotype is specified (nd = not determined), and the target rank in the SPIP list of functionally unknown proteins (**Table S6**).

**Figure 4:**
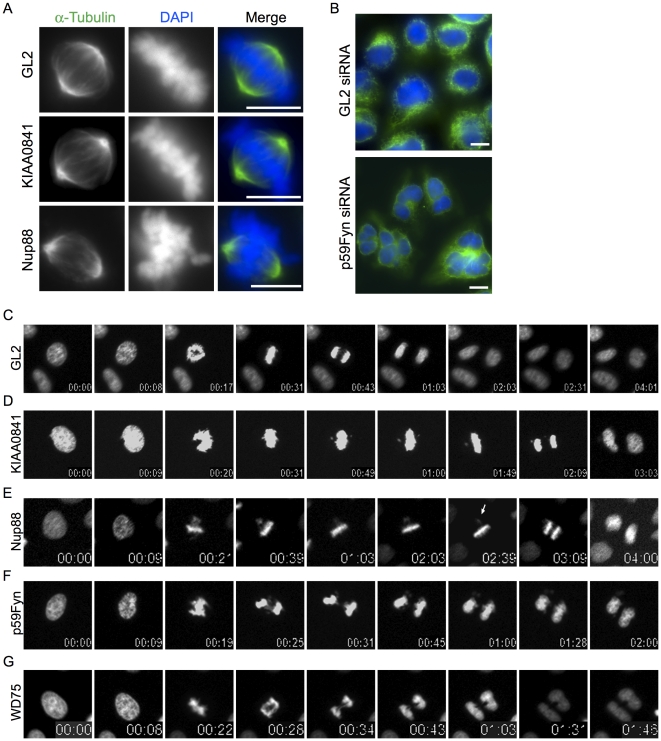
Mitotic phenotype observed upon depletion by siRNA of the selected predicted spindle proteins. (**A**) HeLa S3 cells were treated for 48 h with control (GL2) or *KIAA0841*-and *Nup88* specific siRNAs, respectively, then fixed and stained with α-Tubulin (green). DNA was visualized using DAPI (blue). Bar = 10 µm. (**B**) HeLa S3 cells were treated for 48 h with control (*GL2*) and p59Fyn specific siRNAs, respectively, then fixed and stained with α-Tubulin (green). DNA was visualized using DAPI (blue). Bar = 10 µm. (**C–G**) Stills of representative movies of H2B-GFP expressing HeLa S3 cells treated with control (*GL2*), *KIAA0841*, *Nup88*, *p59Fyn* and *WD75* siRNAs for 36 h before filming. Time points are indicated in h:min.

As a complementary functional approach we investigated a potential mitotic role for the selected proteins using (siRNA)-mediated mRNA knockdowns (**Fig. 4**, **Table 3** and **Tables S7 and S8**). Depletion of most of the analyzed proteins resulted in defects in normal mitotic progression (mitotic delay or faster progression through mitosis, chromosome congression and/or segregation defects and cytokinesis defects) (**Fig. 4**, **Table 3**).

We specifically confirmed a mitotic-related function for several proteins for which, in addition, we described their spindle localization, e.g. GA2L3 (*Gas2L3* [4]) (**Fig. 4A**) and we also confirmed the localization and the mitotic phenotype upon depletion of several proteins that were described as spindle associated during the course of this study, e.g. MORC2 [36] and CCDC99 (later named as *hSpindly*) for which we and others subsequently showed that it functions in the control of kinetochore-associated dynein, spindle orientation and mitotic checkpoint control [37], [38], [39], [40]. Another positive hit in our targets is nucleoporin Nup88 (**Figs. 3** **,** **4A and 4E**), in line with emerging results suggesting that nucleoporins play a role in bipolar spindle assembly [41], [42] and mitotic progression [43]. Furthermore, the abnormalities observed during mitotic progression upon depletion of some of the selected targets localizing to chromosomes (**Fig. S4B**), would also suggest a possible role for these proteins in mitosis. It is also interesting to highlight the faster progression through mitosis observed on depletion of WD75 and p59Fyn (in average 22 min and 14 min from nuclear envelope breakdown (NEBD) to anaphase onset compared to 45 min for control (GL2)-treated cells) (**Fig. 4G**).

Overall, these experimental results affirm the value of our computational framework to guide experimental validation. Four additional proteins have been characterized by other groups in the course of our studies (**Table 3**). KIAA1794, shown to be required for DNA repair [44], EML3 [36], C14Orf106 (M18BP1) [45] and finally, C15Orf23, named recently as SKAP [46], [47], [48].

In summary, 13 of the 14 cloned proteins showed features of localization and/or phenotypic alterations indicative of their true association with the spindle (see **Tables 3** and **S7**). And three of the remaining six not cloned proteins were demonstrated to have spindle localization by other labs (**Table 3**). Mitotic localization was confirmed for 15 of the 20 selected proteins (by us and other labs), including the positive control C1Orf48, and excluding KIAA1794 for which the localization in mitosis was not explored by Smogorzewska and co-workers. For 12 out of 16 the specific localization to the mitotic spindle could be confirmed experimentally.

These experimental validations give a success rate of ∼75% for the experiments guided by SPIP computational predictions. This success rate is clearly better than the ∼35% obtained previously by human expert selection from the Sauer proteomics dataset [2]. Further validation of the SPIP 158 ranked protein list with the Mitocheck phenotypes gives additional support to the experimental validation results, confirming the good performance of the SPIP platform in this dataset (**Text S1 section 6** and **Fig. S3**)

### 4. The spindle interaction network and the detection of “hidden hubs” – poorly characterised proteins with many potentially important interactions

We generated a spindle sub-network and analyzed this to uncover important, missing information on the spindle, revealed by exploring the interactions between known and putative spindle proteins. A protein network is a set of proteins connected by known or predicted protein interactions or associations. By spindle sub-network we mean the network of all the protein pair interactions retrieved from different resources (e.g. protein interactions retrieved from experimental –KG or predicted –PG datasets; see **Methods section 4**) that involve at least one known spindle protein partner.

We searched for highly connected proteins predicted to be more associated with the spindle sub-network in the human interactome, than the rest of the human interactome. The set of known spindle proteins was generated by combining the SEED and EXPERT datasets (see **Methods sections 1** and **4** and **Text S1 sections 1 and 7**, **Table S9 and Figure S10** for a description of the “spindle hidden hubs” ranked results).

**Figure 5:**
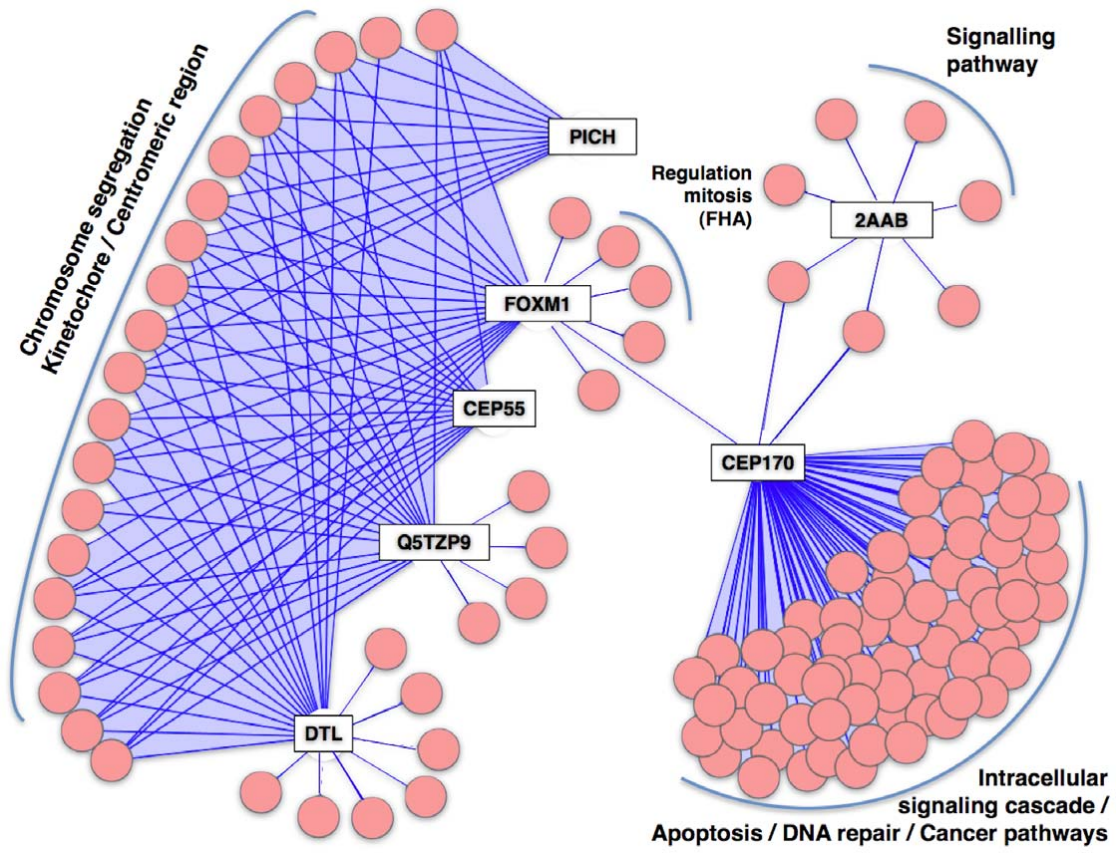
Network model of the hidden spindle hubs. Hidden spindle hubs (rectangular nodes) and associated known spindle proteins (pink circle nodes). Enriched functional classes related to spindle clusters are indicated – see Methods (black labels). For the spindle interacting proteins IDs see **Table S10**.

We analyzed the structure of the spindle sub-network to identify potentially important proteins acting as hubs. Some of the hubs clearly correspond to important spindle proteins with well characterized molecular and cellular functions, not previously believed to have many interaction partners. In these cases, our predictions have revealed their possible actions as highly connected interactors in the spindle sub-network suggesting additional roles for them in the interaction network (**Fig. 5** **and** **Table 4**). Interestingly, many other hubs correspond to proteins for which the current functional characterization is rather poor. We describe these as “hidden spindle hubs”. We focused our analysis on these proteins, and particularly on those that possess a large number of predicted connections to known spindle proteins since they might be particularly interesting (**Table 4**).

**Table 4:** Summary of the results of the ‘*hidden spindle hubs*’ sub-network functional analysis.

Spindle hidden hub clusters	Functional composition	%	Spindle partners IDs
CEP55	Chromosome segregation/kinetochore/	50	O14630; O95229; Q13257; Q15003; Q15021; Q15398; Q4LE75; Q86VS5; Q8NFH4; Q96E58; Q9H900; Q9HBM1; O15392;O94814;P50748; Q8NFU6;O43684
PICH	centromeric region		
DTL			
Q5TZP9			
FOXM1			
FOXM1 (regulation)	Regulation of mitotic cell cycle	38	Q8WV29; Q13257; O95229; Q9H900; **Q96EP1**; Q15398; Q96E58
	FHA: Forkhead regulatory domain		**Q96EP1**; Q15058; O95068
CEP170	Intracellular signaling cascade	25	O00409; **O14757**; **O60229**; P51813; P78317; **Q12933**; **Q13009**; Q13131; Q14676; **Q15052**; Q15118; Q15788; Q86SQ0; **Q96CA5**; **Q9Y4K3**; Q9Y6Q9
	Apoptosis/regulation of apoptosis	19	O43353; **O60229**; P14625; **Q12933**; **Q13009**; **Q13489**; **Q15052**; Q86TM6; **Q96CA5**; Q9H422; Q9NS56; **Q9Y4K3**
	Response to DNA damage stimulus	13	**O14757**; Q5FBX2; Q58F55; Q2TAZ4, A2RRA8; P09874; **P62877**; Q7LGC1; Q12888
	Pathways in cancer (KEGG)	11	Q00987; **Q12933**; Q8NEH5; **Q13489**; Q14568; P14625; **P62877**
2AAB	Signaling pathways	71	O94863; Q8TB43; P42345; Q9Y632; O75620

From right to left: Spindle hidden hub clusters, Spindle hidden hub proteins considered in each cluster; Functional composition; enriched functional classes in each cluster; %, coverage of the functional classes over the total number of interacting spindle proteins; Spindle partners IDs, the accession numbers in Uniprot of the interacting spindle proteins annotated under each enriched functional class. Proteins repeated in different functional classes within the same cluster are labeled in bold.

One of the most interesting cases is a highly integrated cluster with an average of 19 predicted interactions within the spindle sub-network and which includes proteins associated with the kinetochore and chromosome segregation process (**Fig. 5** and **Table 4**) such as: FoxM1, PICH (ERCC6L), Cyclin B1-like protein Q5TZP9, Cep55 and DTL.

PICH, is a Polo-like kinase 1 (Plk1) substrate [49] that concentrates in the centromere/kinetochore (KT) region of mitotic chromosomes and spreads over the chromosome arms in response to Plk1 inactivation. FoxM1 acts as a key transcriptional regulator of G1/S progression and as a key constituent of the G2/M transition [50], [51]. SPIP predictions indicate that FoxM1 seems to perform an important regulatory role since many of the predicted FoxM1 spindle interactors are shown to be involved in mitotic cell cycle regulation (**Table 4**). Recent studies [52] support this regulatory role showing that Plk1-dependent regulation of FoxM1 activity provides a positive-feedback loop ensuring tight regulation of transcriptional networks needed for mitotic progression.

The predicted functional relationship of Cep55 and DTL to kinetochore and chromosome segregation is still unknown. Cep55 is a centrosomal component that localizes to the mother centriole during interphase and whose centrosome dissociation is triggered by Cdk1-dependent phosphorylation upon mitotic entry. It localizes to the midbody and plays a role in cytokinesis [53]. Centrosomes in mammalian cells have recently been implicated in cytokinesis. Therefore, it will be interesting to explore the possible role of Cep55 in the centrosome, chromosome segregation and cytokinesis. DTL, is required for CDT1 proteolysis in response to DNA damage through the CUL4-DDB1 E3 ubiquitin-protein ligase. DTL seems to be necessary to ensure proper cell cycle regulation of DNA replication. The predicted spindle partners suggest that DTL plays a mitotic functional role related to the kinetochore chromosome attachment process [54] [55].

Another interesting spindle hub-protein is Cep170 with 72 predicted spindle interaction partners, which puts it at the top of the “*spindle hidden spindle hubs*” ranked list (**Table 4**). Cep170 is a centrosomal protein that plays a role in microtubule organization [56]. During mitosis, it localizes to the spindle microtubules near the centrosome and maintains correct organization of the MTs at the spindle pole. Cep170 is phosphorylated by Plk1 [56] and acts as a marker for maternal centrioles [57]. SPIP predictions indicate that this cluster could be part of an important signaling pathway that is yet to be elucidated (see **Fig. 5** and **Table 4**).

Finally, it is worth mentioning the PPP2R1B protein, which corresponds to the 65 kDa regulatory subunit A of the serine/threonine-protein phosphatase 2A. The regulatory nature of this sub-unit is supported by the predictions, with 71% of the predicted spindle partners involved in signalling pathways (**Fig. 5**). PPA2 phosphatase has been associated with the kinetochore/spindle checkpoint regulatory pathway in yeast and localises at centromeres probably protecting eukaryotic centromeric regions.

## Discussion

Spindle-associated proteins cover a broad range of functional categories as they can be mechanical and structural components; cargo proteins transported by the spindle apparatus; as well as proteins involved in the regulation of spindle assembly. Capturing this complexity poses a great challenge for any type of experiment, particularly since some of the high throughput technologies provide only indirect evidence about molecular functions, a situation particularly acute for siRNA experiments. To capture more of this large functional space we developed and validated, using multiple approaches, the SPIP platform, a computational method based on the integration of a variety of orthogonal methods ranging from Neural Networks to analysis of co-occurrences in publications.

We assessed the function of a number of novel predicted candidate spindle proteins to demonstrate that this computational methodology significantly improves the chances of selecting true spindle proteins and is better than a manual exploration [2]. About 75% of the proteins selected for experimental verification were validated by co-localization and/or interference experiments or more sophisticated approaches. This success rate is much higher than the 35% previously obtained by the manual curation of the potential candidates. The success rate is even more significant considering that the predictions were done on the pool of proteins remaining after human experts had picked the most obvious candidates for their first experimental analysis [2], and represents a further demonstration of the usefulness of the SPIP computational strategy (see **Text S1 section 3** for the details of this comparison).

Our experiments confirmed the mitotic localizations of 16 of the 20 selected proteins (including the positive control C1Orf48, and excluding KIAA1794 for which the localization in mitosis was not explored by Smogorzewska and co-workers [44]. For 11 out of 16 the localization to the mitotic spindle could be confirmed experimentally. Among the potential new discoveries we can mention proteins such as: GA2L3, MORC2 and CCDC99 that in some cases have already been confirmed by more direct experimental approaches (i. e. CCDC99 or hSpindly [37], [38], [39], [40]). Furthermore, the abnormalities observed during progression through mitosis on depletion of some of the selected targets, that localized to chromosomes (WD76 and WD75 and Pescadillo homologue 1), would suggest a possible role of these proteins in mitosis, increasing the number of true positives in our candidate list. It is also interesting to highlight the faster progression through mitosis observed on depletion of p59Fyn. Overall, these experimental results affirm the potential applicability of our computational framework to assist experimental validation.

To complement the characterization of potential targets we analyzed the set of putative spindle-associated proteins by considering the network of interactions they participate in. In summary, the connectivity of the “hidden spindle hubs” in the spindle sub-network suggests that they have a role in spindle formation and/or regulation that was not previously suspected (e.g. Cep55), or in other cases represents the discovery of new associations with the spindle system, e.g. Cyclin B1-like protein Q5TZP9. This is for instance well reflected by the relationship between Cep55 and FoxM1 (i.e. siRNA-mediated depletion of Cep55A alters the expression of FoxM1 [58]). Results obtained by the Mitocheck consortium revealed clear alteration in the mitotic phenotypes obtained upon depletion of several of the putative hidden spindle hubs (for instance Cep55, DTL, cyclin B1 etc.). A common feature of many of the predicted “hidden spindle hubs” is their implication in transient regulatory and signalling interactions, i.e. FoxM1, PICH; cyclin B1-like, Cep170 and 2AAB, which may explain why many of the predicted interactions have not been detected by conventional experimental approaches, such as, for example, high-throughput Y2H assays [59].

Our results suggest we are still far from knowing the complete repertoire of functionally important components of the spindle. However, our SPIP platform has provided many predicted components which are potentially reliable and which would be a considerable aid in guiding any further experimental effort. In particular, these predictions may help us to fill in gaps in functional space that remain elusive to high-throughput approaches, i.e. transient interactions (see [59]). Our study shows that integrated bio-computational approaches followed by experimental validation of individual proteins are key to exploring these hidden regions in protein networks.

## Materials and Methods

### 1. The protein data sets

The original proteomics data set from Sauer *et al.* [2] was mapped (**Table S10**) to the UniProt database (Uniprot KB/Swiss-Prot release 56.0 of 22 July 2008) primary accession entries. The human proteins were obtained from the same UniProt release. The SEED dataset is composed of 149 known spindle proteins from the Sauer set (**Table S10**). The EXPERT set is a manually generated data set of spindle proteins, obtained by manually checking publications selected with the help of a bioinformatics system (see **Text S1 section 1** and **Fig. S5**).

### 2.1 Assessing the Results of the individual methods

All the methods described above were run against all sequences in the human proteome file (see data sets, **section 1**). Methods predicting protein pairs can retrieve the same target protein associated with different bait proteins (SEED spindle proteins) with different prediction scores. In order to transform the bait-target predicted lists into target prediction lists, targets from every bait-target list of predictions were scored by the best score out of all the pairs in which the target was detected. The number of predictions generated by each method is shown in **Table 5**.

**Table 5:** Number of predictions retrieved by each method.

Method	# predictions
COCITE	1,982
MLNN	19,770
hiPPI	1,218
CODAcath	11,949
CODApfam	13,468
DORA	5,619
GECO	7,746
GOSS	6,695

### 2.2 Integrating the Methods

**Calculation of P-values**, a cumulative frequency distribution is calculated for the scores of each of the prediction methods (COCITE, GECO, hiPPI, etc.). The partial/single Probability Density Functions (PDF) associated with the score distributions, for each method, is calculated using the curvefit tool from MATLAB in order to translate the scores into p-values. Since Fisher's integration method formula has a chi-squared distribution which requires a sum of independent normal distributions, we carried out right tailed Ztests (at p = 0.05 significance level) to ensure that the P-values PDF distributions follow independent standard normal random variables.

**Data integration using Fisher**, the prediction p-values obtained for each method were integrated using the Fisher statistics method [60], [61]. If a protein contains more than one domain predicted by DORA, the protein-domain prediction with the best score is selected, amongst all the predictions, and integrated by SPIP. Statistical Dependence between the prediction datasets was calculated with mutual information statistics (**Tables S3 and S4**). MI is a metric that quantifies the difference in the ratio of the observed joint distribution of X and Y and the expected joint distribution, assuming X and Y are independent (H_0_ null hypothesis; see **Text S1 section 2** for MI calculation details). We calculated the D normalised values of the MI values based on the entropy (H) of each pair of prediction sets compared in Tables S3 and S4. Let H be the entropy between X and Y samples and I the corresponding mutual information. Then, the expression d(X,Y) = H(X,Y)−I(X;Y) meets the basic properties of a metric (H tends to be about maximum and model the samples X and Y as independent); most importantly, the triangle inequality, but also non-negativity and symmetry. In addition, one also has, d(X,Y)≤H(X,Y), and so obtains D(X,Y) = d(X,Y)/H(X,Y)≤1. In this way, D is a normalised MI-based metric that indicates the probability to reject the H_0_ hypothesis (X and Y are independent) being false. *D* is a universal metric, in that if any other distance measure places X and Y close, then *D* will also consider them close. We need a universal metric to ensure that the MI results do not depend on the metric selected [62].

*D* is a metric as *d* because when considering conditional entropy we realize that we are able to draw upon a set-theoretic vision of information such as D(X,Y) = 1−I(X;Y)/H(X,Y), which meets the Jaccard distance between X and Y. In this way, *D* is a normalised MI-based metric as *d* [63].

**Calculation of the ROC and PR curve**s: Sensitivity (also called Recall; TP/TP+FN), Specificity (TN/TN+FP); and Precision (TP/TP+FP) were calculated using the 73 EXPERT dataset as True Positives (TP), and random datasets as True Negative (TN) sets. False Negatives (FN) were calculated as the # of TPs predicted as TNs; and False Positives (FP) were calculated as the # of TNs predicted as TPs along the ranked lists.

### 3.1. Criteria for selecting genes for experimental validation

A set of 20 target proteins was selected applying the same general criteria as applied in the original Sauer selection protocol. Using these criteria we excluded: i) proteins of more than 150 kDa that were technically difficult to clone and express [64], [65] ii) proteins with a predicted cellular localization unlikely to be spindle associated e.g. mitochondrial proteins; iii) proteins assigned to functional classes less frequently predicted to be involved in spindle function, e.g. mitochondrial proteins.

### 3.2 Plasmid generation

Candidate selected genes were amplified by PCR from commercially available cDNA clones from the “Deutsches Ressourcenzentrum fur Genomforschung” (RZPD) using sequence-specific sense and antisense primers. The ORFs (open reading frames) that were not commercially available were cloned by direct PCR using a HeLa or testis cDNA library as template. Myc-constructs were generated by inserting the whole coding region of each cDNA in frame into an N-terminal 3xMyc-pCDNA3.1 vector (Invitrogen). Authenticity of all constructs was verified by DNA sequencing.

### 3.3. Cell culture

HeLa S3 cells [66] were grown at 37°C under 5% CO_2_ in DMEM (Invitrogen), supplemented with 10% FCS and penicillin-streptomycin (100 U ml^−1^ and 100 µg ml^−1^, respectively).

### 3.4. Plasmid transfection

Transient transfection of HeLa S3 with plasmid DNA was performed with TRansl®-LT1 reagent following the manufacturer's recommendations (Mirus Bio Corporation). After 12 h cells were arrested with thymidine and 12 h later they were released into fresh medium, allowing them to accumulate in mitosis (ca. 36 h in total).

### 3.5. siRNA transfection

All siRNAs were synthetic double-stranded stealth select oligos (Qiagen) (**Table S8**). SiRNA duplexes were transfected using Oligofectamine (Invitrogen) as described elsewhere [67]. As a control, a duplex (GL2) targeting luciferase was used.

### 3.6. Immunofluorescence (IF) microscopy

Cells were grown on coverslips and fixed and permeabilized as described previously [68]. Primary antibodies used in this study were mouse mAb anti-Myc (1∶10, 9E10 tissue culture supernatant), sheep mAb anti-alpha-tubulin (1∶250, Santa-Cruz Biotechnology) and human CREST autoimmune serum (1∶500, Immunovision). Primary antibodies were detected with Alexa-Fluor-488 and Alexa-Fluor-555-conjugated goat anti-mouse, anti-rabbit or anti-goat IgGs (1∶1000, Molecular Probes), respectively. DNA was stained with 4′6-diamidino-2-phenylindole (DAPI, 2 µg ml^−1^). Immunofluorescence microscopy was performed using a Zeiss Axioplan II microscope (Zeiss) with Apochromat 40× and 63× oil immersion objectives, as described before [68].

### 3.7. Live-cell imaging

For live-cell imaging, a HeLa S3 cell line stably expressing histone H2B-GFP was used [68]. Cells were treated with siRNAs for 36 hours, before changing the medium to CO_2_ independent medium, and the culture dish was placed onto a heated sample stage within a heated chamber (37°C). Live-cell imaging was performed using a Zeiss Axiovert 2 microscope equipped with a Plan Neofluar 20× objective. Metaview software (Visitron Systems GmbH) was used to collect and process data. Images were captured with 5 ms (GFP) exposure times with 3 min intervals for 16 hr.

### 4. Calculation and ranking of the spindle hidden hubs

In order to predict hidden spindle hub proteins we first constructed a spindle sub-network of the human interactome based on a set of known proteins obtained by combining the 149 known spindle proteins from the Sauer set (SEED) with the list of 73 EXPERT proteins (giving a total of 223 curated spindle proteins).

We also assembled two independent protein-protein interaction networks in the whole human proteome (1) from a combination of the experimental datasets - ‘Knowledgegram’ (KG) and (2) from a combination of all predicted datasets - Predictogram (PG). The KG dataset combines all the experimental PPI data from the following databases: Reactome, Kegg, GO (Using the GOSS method), FunCat, Intact, MINT and HRPD. The PG contains the sum of the predicted PPI data generated by the pure *ab-initio* methods in SPIP: GECO, hiPPI, CODAcath and CODApfam datasets. We selected predictions with p_values< = 0.014, a threshold that we have identified from benchmarking against gold standards performs with a precision ≥80% [27].

We then took the top 2% of proteins (642 proteins) from the SPIPall ranked list on the human proteome, as representative of highly probable spindle associated proteins and calculated the number of connections (ki) (in both the KG and the PG) between the predicted proteins (top-2% of SPIPall) and the 223 known proteins in the spindle sub-network and also between the predicted proteins and the human proteome.

For each protein i, compute its degree k_i (KG) in the KG dataset and its degree k_i (PG) in the PG dataset. We then identified and ranked ‘hidden’ spindle hubs as those targets with low ki values in the KG dataset (KG_ki) but high ki values in the PG (PG_ki) and with a high percentage of the ki connections specific to spindle partners i.e. the 223 known spindle sub-network set (% spindle_specific in **Table S9**).

The rationale of this selection criteria was to select “hidden hub” proteins ie those proteins with very few experimentally known interactions reported in KG (low ki value in the KG network –KG_ki) but many predicted interactions in PG (high ki value in the PG network –PG_ki; **Table S9**) i.e. at least five times more interactions in the PG. In addition, “hidden hubs” specific to the spindle system should have a high percentage of their connections to proteins belonging to the spindle sub-network. These criteria were chosen as they were strict enough to give proteins that were likely to be hidden hubs but gave a reasonable number of predictions and not too many as to prevent careful manual evaluation. Functional annotation of the spindle hidden hubs was performed using the literature and the DAVID Server. (more details in **Text S1 section 7**).

## Supporting Information

Figure S1 **Validation of the LM, NNI and DGC methods.** Test of the performance of the pair-wise combination of methods using the text mined, manually curated gold standard dataset - EXPERT.
(DOCX)

Figure S2 **Enrichment in Mitocheck phenotypes in the human proteome SPIPall ranked list.**
(DOCX)

Figure S3 **Mitocheck genes and phenotypes distribution in the SPIP158 unknown protein ranked list.**
(DOCX)

Figure S4 **Mitotic localization of selected predicted candidate spindle proteins.**
(DOCX)

Figure S5 **The literature dataset.**
(DOCX)

Figure S6 **The COCITE scoring system.**
(DOCX)

Figure S7 **The mitotic spindle predictor.**
(DOCX)

Figure S8 **ROC analysis for COCITE method.**
(DOCX)

Figure S9 **Random test for the analysis of the statistical significance of the Mitocheck enrichments.**
(PDF)

Figure S10 **Non-hub hidden spindle proteins analysis.**
(DOC)

Table S1 **Whole human proteome predictions. (Large file).**
(TXT)

Table S2 **Calculation of the area under the ROC curves to measure and compare the statistical significance of the methods performance.**
(DOC)

Table S3 **Conditional independence measures of the three types of spindle prediction datasets.**
(DOC)

Table S4 **Study of dependencies amongst the individual prediction methods.**
(DOC)

Table S5 **Top 250 proteins in SPIPall with annotations related to mitotic function/spindle localization. (Large file).**
(XLS)

Table S6 **Ranked list of proteins classified as “functionally unknown” by Sauer et al. (Large file).**
(XLS)

Table S7 **Summary of the mitotic phenotype observed upon depletion by siRNA of the selected predicted spindle proteins.**
(DOCX)

Table S8 **Specific siRNA oligonucleotides sequences used in this study.**
(DOCX)

Table S9 **Ranked list of predicted spindle hidden hubs. (Large file).**
(TXT)

Table S10 **Datasets used in this study. (Large file).**
(TXT)

Table S11 **Results of the Runstest scores run for the all-Mitocheck phenotypes rank.**
(DOC)

Text S1 **Supporting Materials and Methods.**
(DOC)
