# Supplementary material for: Uncovering the Molecular Machinery of the Human Spindle—An Integration of Wet and Dry Systems Biology
Source: PLoS One. 2012 Mar 9;7(3):e31813. doi: 10.1371/journal.pone.0031813 (PMC3302876; doi:10.1371/journal.pone.0031813)
Supplement: Figure S1 — Validation of the LM, NNI and DGC methods. Test of the performance of the pair-wise combination of methods using the text mined, manually curated gold standard dataset - EXPERT. (DOCX) [file pone.0031813.s001.docx]

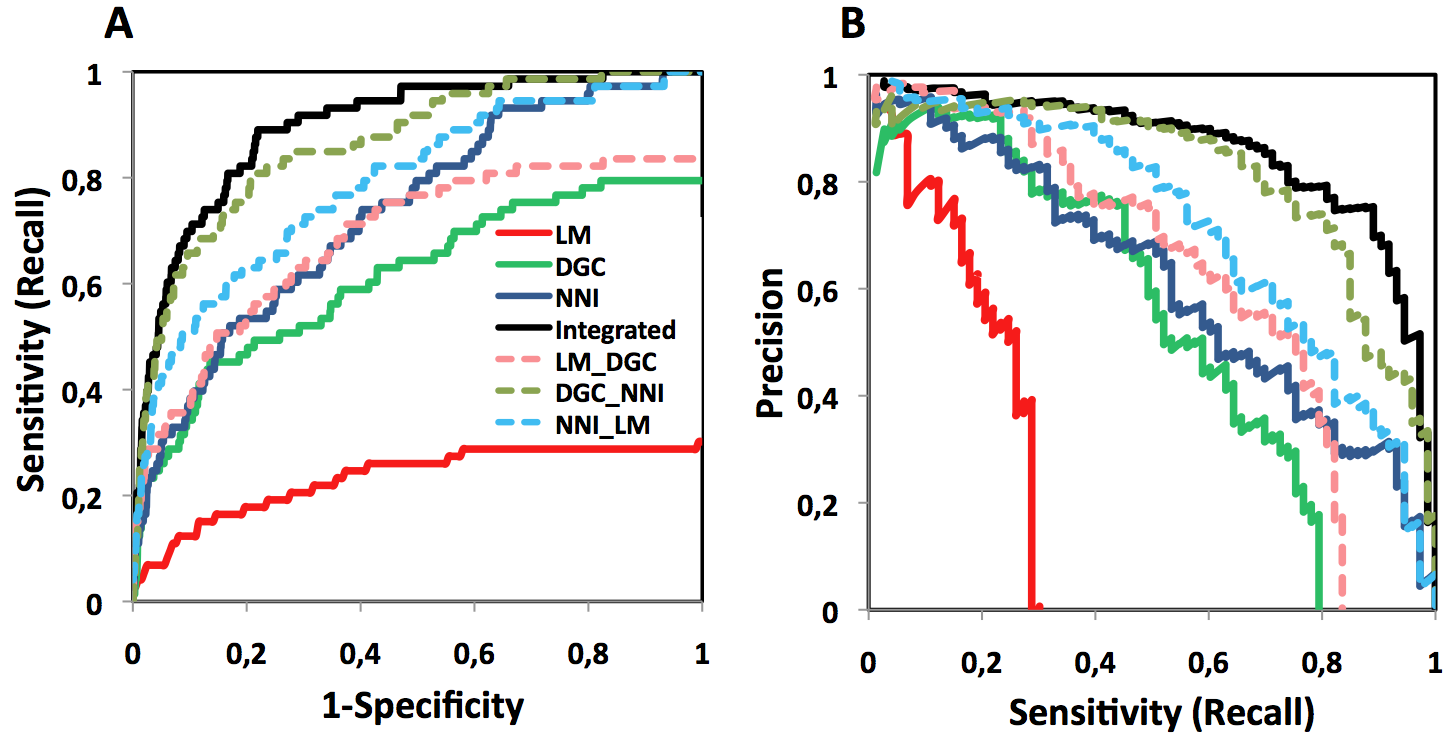


**Supplementary Figure S1**. **Validation of the LM, NNI and DGC methods.** Validation of the pair-wise combination of methods against the text mined, manually curate dataset EXPERT gold standard, used as true positives. The DGC_NNI, NNI_LM and LM_DGC combinations are shown together with the single LM, NNI and DGC predictions and the integration of all of them. (A) ROC curves: Sensitivity (also called Recall; y-axis) versus 1-Specificity (x-axis). And (B) PR curves: Precision (y-axis) versus Recall (x-axis) retrieved by each method and their pairwise combination.
