# Supplementary material for: Uncovering the Molecular Machinery of the Human Spindle—An Integration of Wet and Dry Systems Biology
Source: PLoS One. 2012 Mar 9;7(3):e31813. doi: 10.1371/journal.pone.0031813 (PMC3302876; doi:10.1371/journal.pone.0031813)
Supplement: Figure S2 — Enrichment in Mitocheck phenotypes in the human proteome SPIPall ranked list. (DOCX) [file pone.0031813.s002.docx]

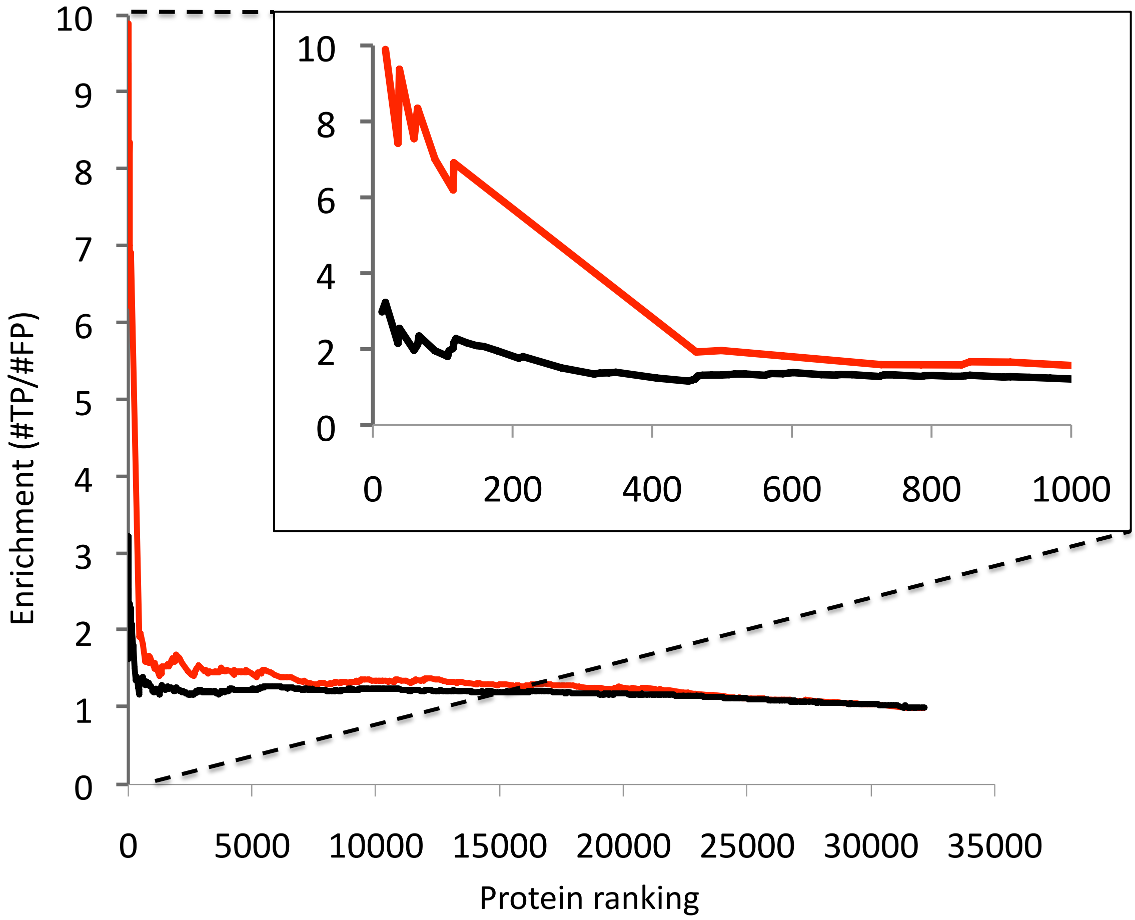

Supplementary Figure S2. Enrichment in Mitocheck phenotypes in the human proteome SPIPall ranked list. The plot shows the Enrichment (TP/FP ratio) versus the ranking of the predicted proteins for all the proteins with a significant phenotype in the Mitocheck dataset (black line) and in the subset of Mitocheck proteins with a spindle related phenotypes (red line). The inset shows the enlarged image of the first 1.000 rank positions' enrichment distribution.
