# Supplementary material for: Uncovering the Molecular Machinery of the Human Spindle—An Integration of Wet and Dry Systems Biology
Source: PLoS One. 2012 Mar 9;7(3):e31813. doi: 10.1371/journal.pone.0031813 (PMC3302876; doi:10.1371/journal.pone.0031813)
Supplement: Figure S3 — Mitocheck genes and phenotypes distribution in the SPIP158 unknown protein ranked list. (DOCX) [file pone.0031813.s003.docx]

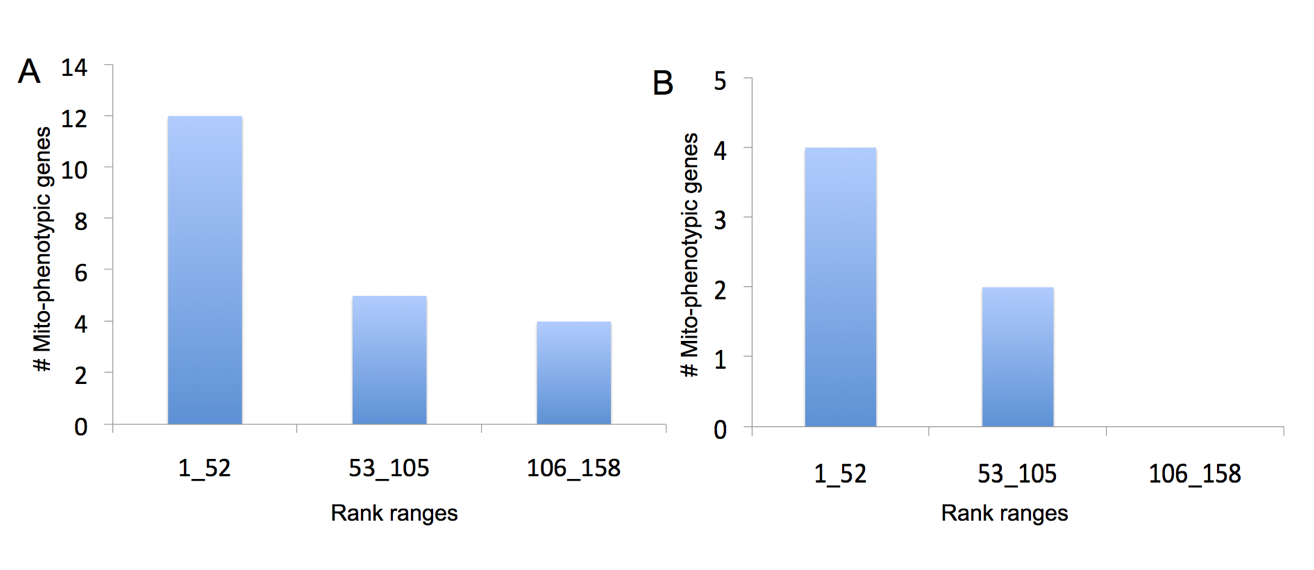


**Supplementary Figure S3**. **Mitocheck genes and phenotypes distribution in the SPIP158 unknown protein ranked list**. (A), number of Mitocheck genes with phenotypes found in the first, second and third 1/3 of the ranked list; (B), the same as panel A but for the Mitocheck spindle related phenotypes
