# Supplementary material for: Uncovering the Molecular Machinery of the Human Spindle—An Integration of Wet and Dry Systems Biology
Source: PLoS One. 2012 Mar 9;7(3):e31813. doi: 10.1371/journal.pone.0031813 (PMC3302876; doi:10.1371/journal.pone.0031813)
Supplement: Figure S4 — Mitotic localization of selected predicted candidate spindle proteins. (DOCX) [file pone.0031813.s004.docx]

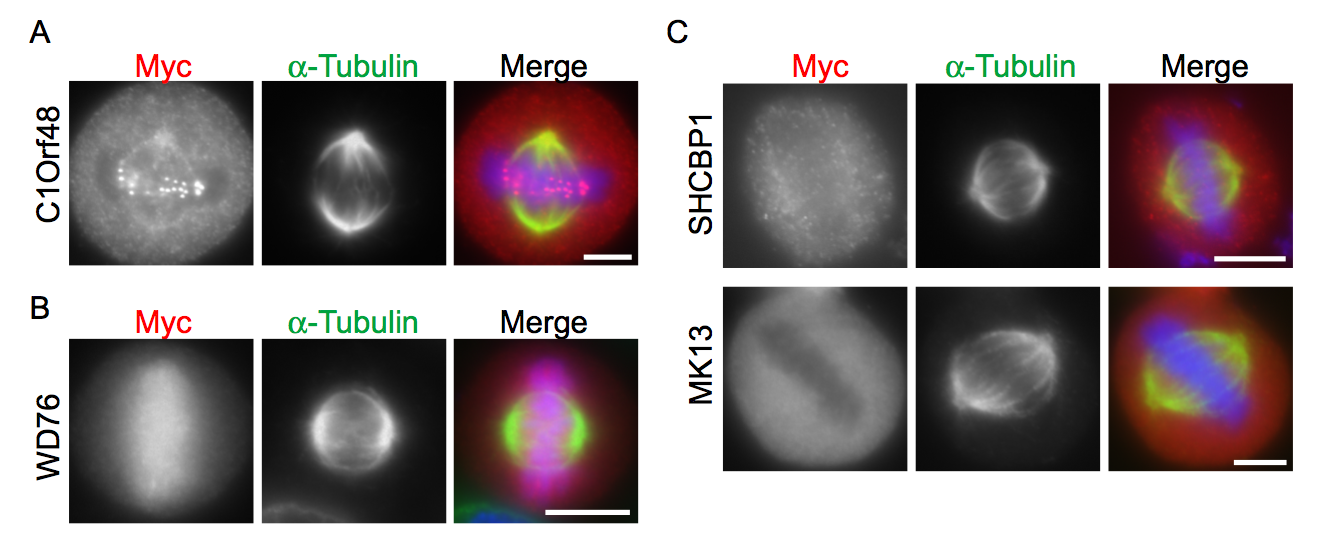


**Supplementary Figure S4. Mitotic localization of selected predicted candidate spindle proteins**. (A- C) HeLa S3 cells were transfected with the indicated myc-tagged constructs, fixed and analyzed by indirect immunofluorescence. Cells were stained with 9E10 anti-myc antibody (red) and with α-Tubulin (green). DNA was visualized using DAPI (blue). Bar = 10 µm.
