# Supplementary material for: Uncovering the Molecular Machinery of the Human Spindle—An Integration of Wet and Dry Systems Biology
Source: PLoS One. 2012 Mar 9;7(3):e31813. doi: 10.1371/journal.pone.0031813 (PMC3302876; doi:10.1371/journal.pone.0031813)
Supplement: Figure S5 — The literature dataset. (DOCX) [file pone.0031813.s005.docx]

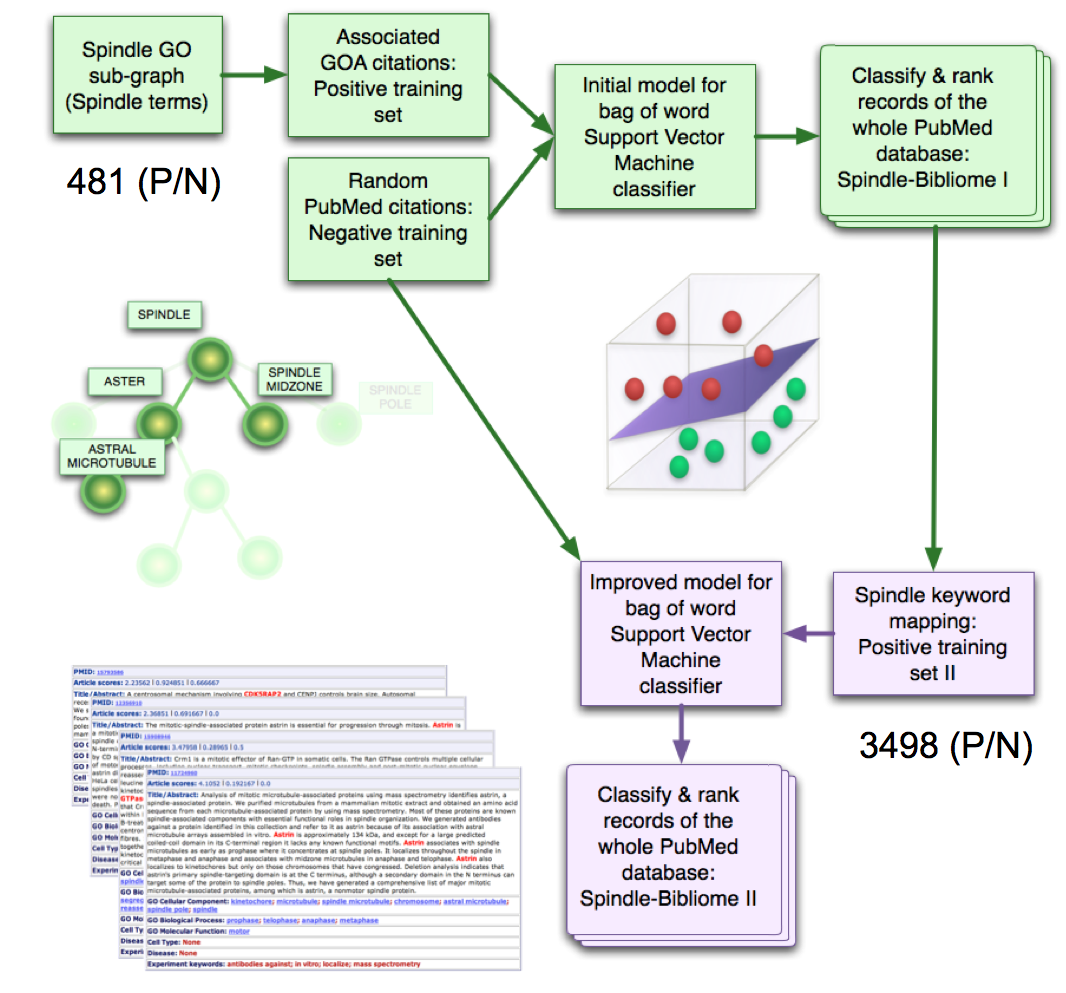


**Supplementary Figure S5. The literature dataset.** Construction of the spindle bibliome. This figure illustrates how a collection of automatically classified and scored articles relevant to the spindle were generated using machine learning techniques based on Support Vector Machines (SVMs). Gene Ontology terms related to spindle pole and their child nodes (GO-subgraph) were initially selected. Articles which had been used to annotate gene products with these terms were derived from GOA, resulting in a small initial positive training set. An equally sized negative set was derived by selecting a random subset of articles from the PubMed database. These two labeled abstract collections served to train an initial model used to classify the entire PubMed database. Articles that had been scored as positive (i.e. spindle relevant) from this initial step and which also mentioned spindle keywords were used as a larger positive training set for re-classification of the PubMed database. The second step classification was also carried out using an SVM classifier using BOW with linear kernel function.
