# Supplementary material for: Uncovering the Molecular Machinery of the Human Spindle—An Integration of Wet and Dry Systems Biology
Source: PLoS One. 2012 Mar 9;7(3):e31813. doi: 10.1371/journal.pone.0031813 (PMC3302876; doi:10.1371/journal.pone.0031813)
Supplement: Figure S6 — The COCITE scoring system. (DOCX) [file pone.0031813.s006.docx]

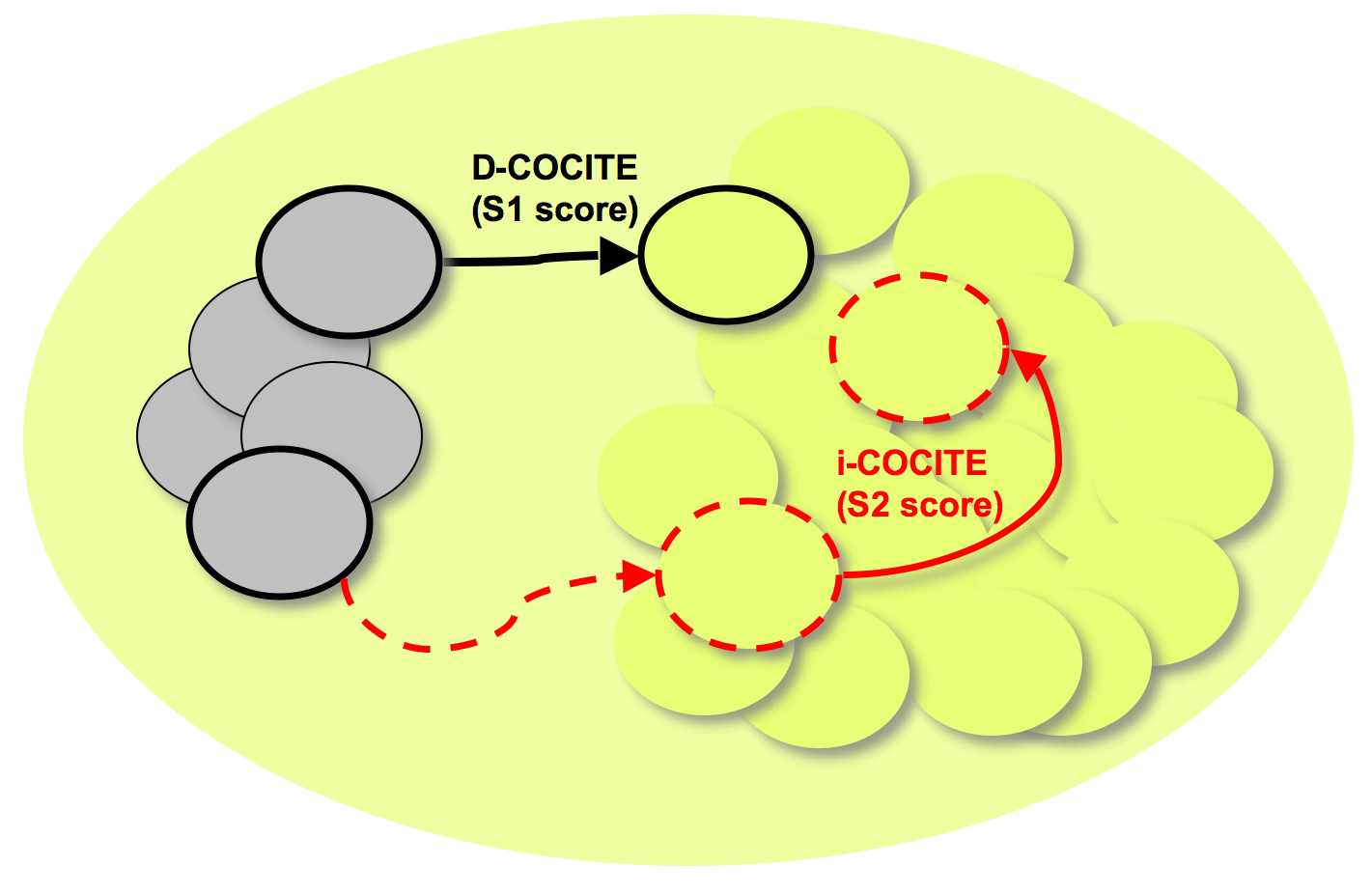


**Supplementary Figure S6. The COCITE scoring system.** The SEED dataset are represented by gray and black solid circles, and are the true experimentally validated spindle proteins in Sauer’s dataset [[1](#_ENREF_1)]. The other groups are human proteins included in the SwissProt database that were also isolated in Sauer’s experiment. The dark brown arrow indicates direct interactions (S1 score, dCOCITE, Supplementary Methods) of a true spindle protein (SEED proteins) with any protein of the proteome (brown solid line circle). There are a total of 102 direct interactions. The red dashed arrows indicate indirect interactions (S2 score, iCOCITE, see Supplementary Methods) of a given true spindle (SEED) protein with a non-spindle protein via an intermediate non-spindle protein (dashed circles). There are 13,940 indirect interactions. The green background represents the whole human proteome (Methods and Supplementary Methods).
