# Supplementary material for: Uncovering the Molecular Machinery of the Human Spindle—An Integration of Wet and Dry Systems Biology
Source: PLoS One. 2012 Mar 9;7(3):e31813. doi: 10.1371/journal.pone.0031813 (PMC3302876; doi:10.1371/journal.pone.0031813)
Supplement: Figure S7 — The mitotic spindle predictor. (DOCX) [file pone.0031813.s007.docx]

**
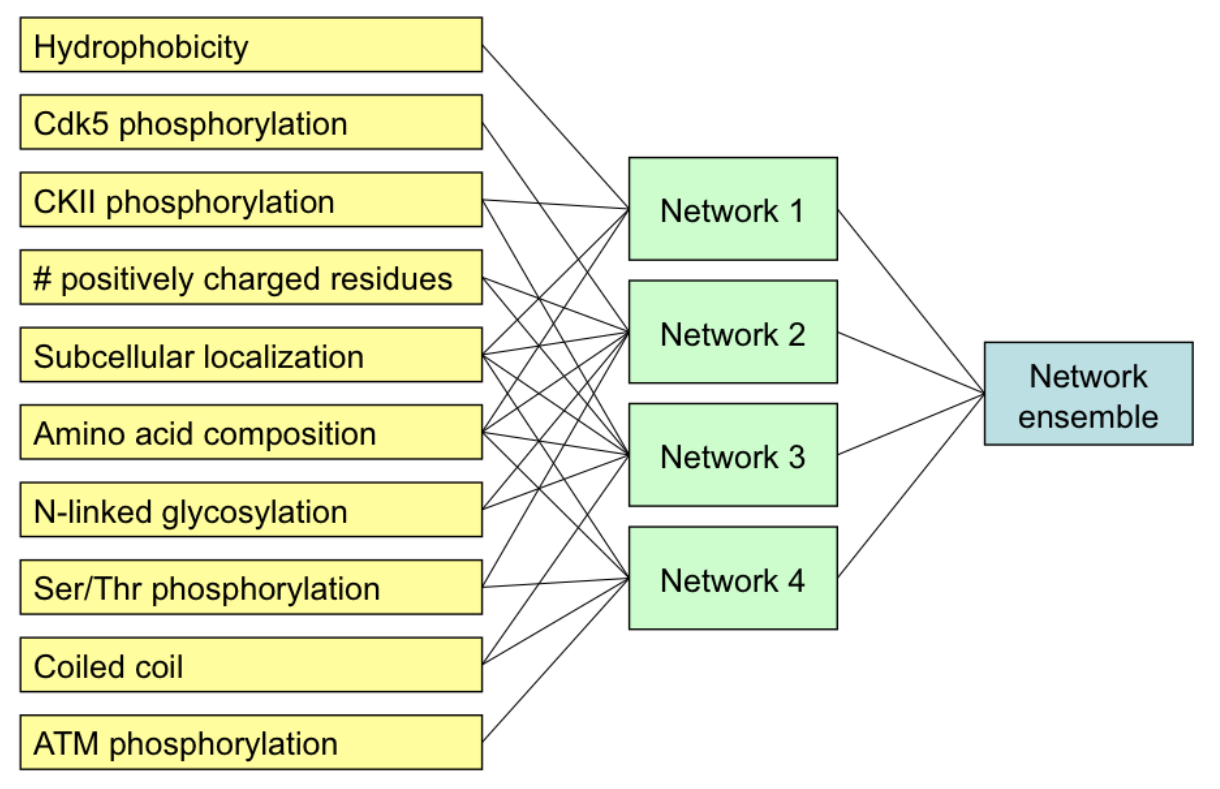
**

**Supplementary Figure S7. The mitotic spindle predictor.** Updated version of the mitotic spindle predictor based on 2nd generation data set and expanded feature list, including custom made coil-coil features (Methods and Supplementary Methods).
