# Supplementary material for: Uncovering the Molecular Machinery of the Human Spindle—An Integration of Wet and Dry Systems Biology
Source: PLoS One. 2012 Mar 9;7(3):e31813. doi: 10.1371/journal.pone.0031813 (PMC3302876; doi:10.1371/journal.pone.0031813)
Supplement: Figure S8 — ROC analysis for COCITE method. (DOCX) [file pone.0031813.s008.docx]

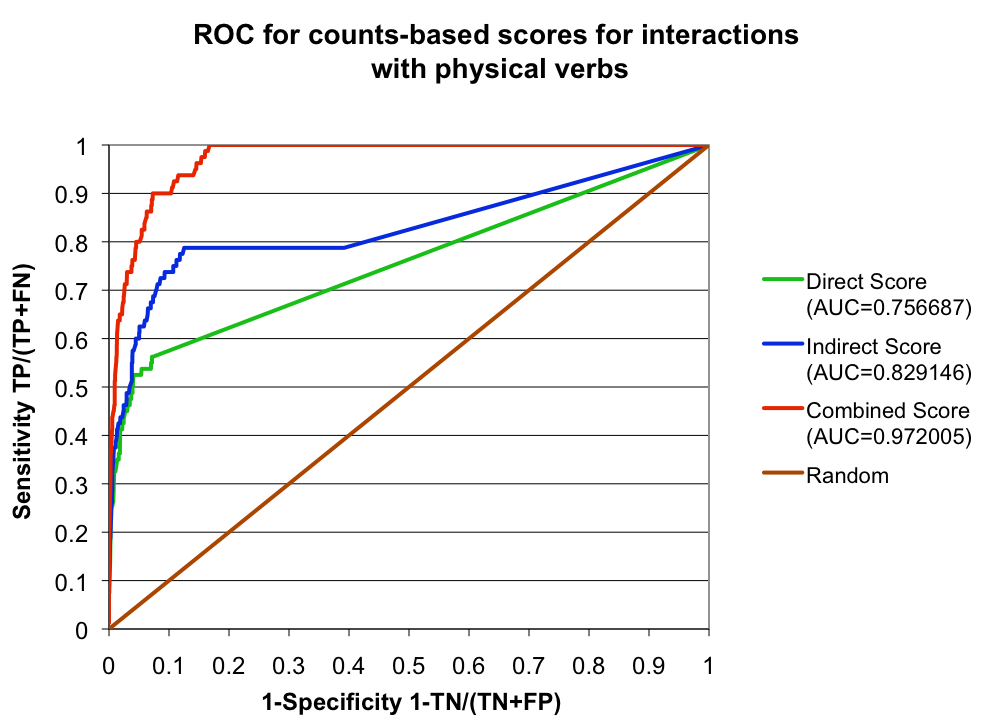


**Figure S8**. **ROC analysis for COCITE method.** The curves are constructed using the whole list of proteins (4,918 proteins) in the COCITE network. The whole list of proteins is sorted according to the scores of the method with the SEED as the reference set: S1 (green line); S2 (blue line) and St (red line). The corresponding AUC values are shown in the figure legend.
