# Supplementary material for: Uncovering the Molecular Machinery of the Human Spindle—An Integration of Wet and Dry Systems Biology
Source: PLoS One. 2012 Mar 9;7(3):e31813. doi: 10.1371/journal.pone.0031813 (PMC3302876; doi:10.1371/journal.pone.0031813)
Supplement: Figure S9 — Random test for the analysis of the statistical significance of the Mitocheck enrichments. (PDF) [file pone.0031813.s009.pdf]

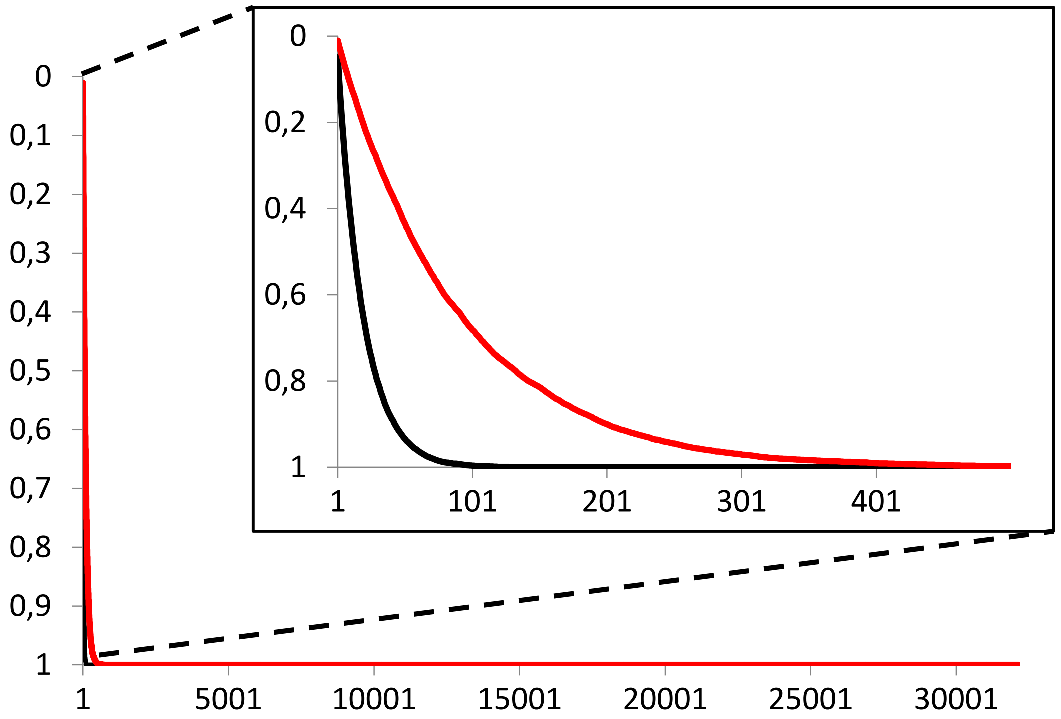

Figure S9. Random test for the analysis of the statistical significance of the Mitocheck enrichments. Random probability (x axis) to obtain TP/FP enrichment ratios equal to or greater than those observed in each of the two Mitocheck ranked lists: the all-Mitocheck dataset: black line; and the spindle related subset: red line. The inset shows the enlarged image of the probability distributions for the first 500 ranked positions.
