# Supplementary material for: Uncovering the Molecular Machinery of the Human Spindle—An Integration of Wet and Dry Systems Biology
Source: PLoS One. 2012 Mar 9;7(3):e31813. doi: 10.1371/journal.pone.0031813 (PMC3302876; doi:10.1371/journal.pone.0031813)
Supplement: Figure S10 — Non-hub hidden spindle proteins analysis. (DOC) [file pone.0031813.s010.doc]

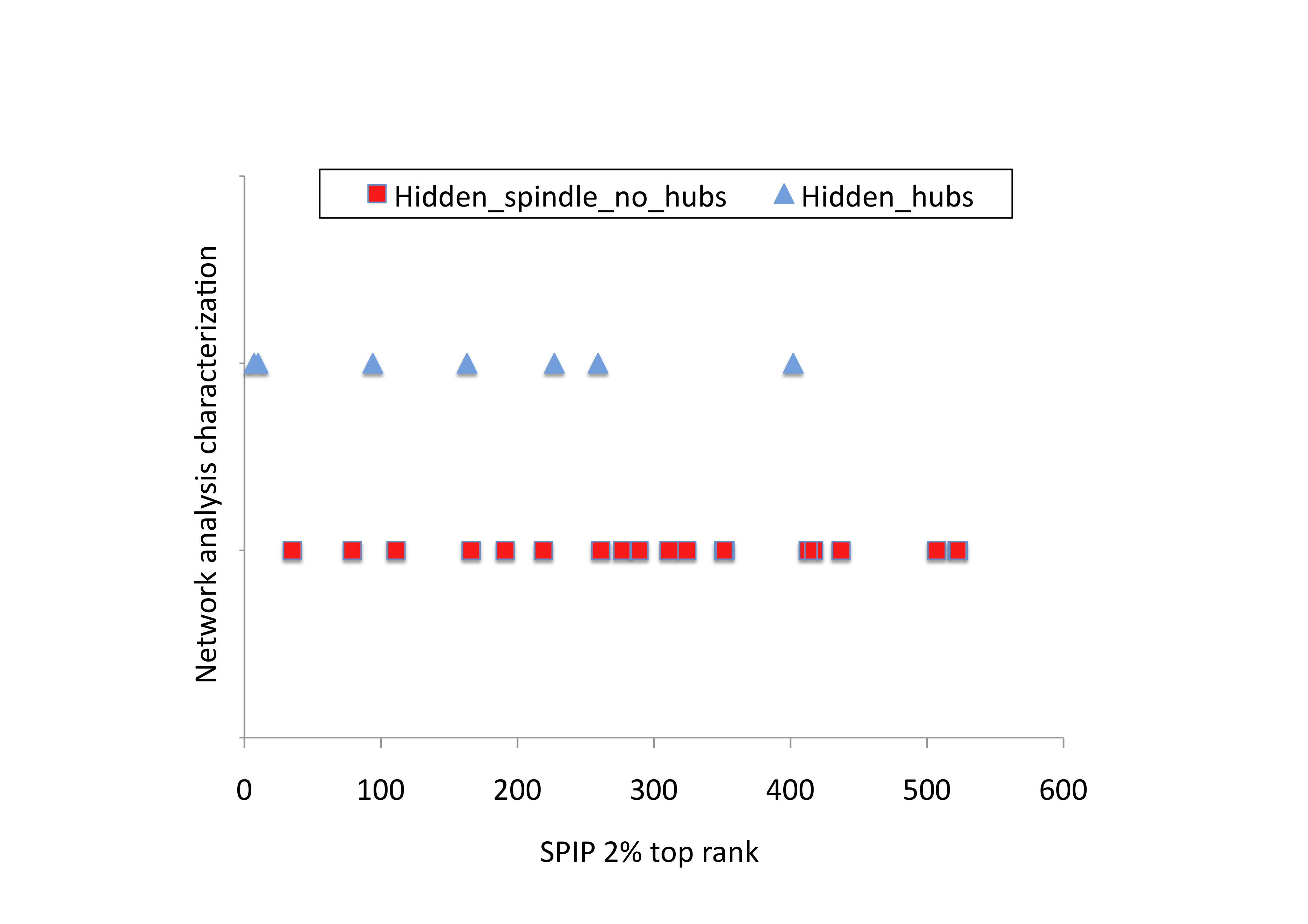


**Supplementary Figure S10. Non-hub hidden spindle proteins analysis**. Position in the SPIP top 2% of ranked predictions (x axis) for the “non-hub hidden spindle” proteins (red square labels) and the “hidden spindle hub” proteins (blue triangle labels).
