# Supplementary material for: Uncovering the Molecular Machinery of the Human Spindle—An Integration of Wet and Dry Systems Biology
Source: PLoS One. 2012 Mar 9;7(3):e31813. doi: 10.1371/journal.pone.0031813 (PMC3302876; doi:10.1371/journal.pone.0031813)
Supplement: Table S2 — Calculation of the area under the ROC curves to measure and compare the statistical significance of the methods performance. (DOC) [file pone.0031813.s012.doc]

| Curve | Method | SAUC | % of Integrated |
| --- | --- | --- | --- |
| ROC curves  (Fig. 2A and S1A) | LM | 1,25 | 9 |
|  | NNI | 5,60 | 38 |
|  | DGC | 8,63 | 59 |
|  | LM_DGC | 10,57 | 72 |
|  | DGC_NNI | 11,80 | 81 |
|  | NNI_LM | 7,67 | 53 |
|  | Integrated | 14,60 | 100 |
| Precision-Recall (PR) curves  (Fig. 2B and S1B) | LM | 0,54 | 5 |
|  | NNI | 3,25 | 30 |
|  | DGC | 3,59 | 33 |
|  | LM_DGC | 4,35 | 42 |
|  | DGC_NNI | 4,81 | 44 |
|  | NNI_LM | 4,47 | 41 |
|  | Integrated | 10,83 | 100 |

**Supplementary table S2. Calculation of the area under the ROC and PR curves to measure and compare the statistical significance of the methods performance.**For the ROC (upper table) and PR (lower table) curves the SAUC (Standardized Area Under the Curve) was calculated using the random model as a standardization measure (SAUC=(AUC-RMA)/S.E.); where AUC is the Area Under the Curve; RMA is the Random Model Area, and S.E. is the Standard Error associated, see below for calculation details); Ztests on SAUC areas showed in all cases. pvalues below 10^-3^ for rejecting the null hypothesis being true (null hypothesis = SAUC areas being random). The *% of Integrated* column shows the percentage ratio between each method´s SAUC area and the Integrated method in both curves estimation (ROC and PR curves). In all cases individual and pairwise combined methods show *% of Integrated* values below 100% indicating lower performance than the Integrated method.

Standard Error Calculations:

Area_2_=Area_1_^2^; Q_1_=Area_1_/(2-Area_1_); Q_2_=2*Area_2_/(1+Area_1_);

V=(Area_1_*(1-Area_1_)+(lu-1)*(Q_1_-Area_2_)+(lh-1)*(Q_2_-

Area_2_))/(lu*lh);

Serror=realsqrt(V);

Where *Area_1_* is the area under the curve, *lu* and *lh* are the abscissa and ordinate value coordinates respectively in the ROC and PR curves.
