# Supplementary material for: Uncovering the Molecular Machinery of the Human Spindle—An Integration of Wet and Dry Systems Biology
Source: PLoS One. 2012 Mar 9;7(3):e31813. doi: 10.1371/journal.pone.0031813 (PMC3302876; doi:10.1371/journal.pone.0031813)
Supplement: Table S3 — Conditional independence measures of the three types of spindle prediction datasets. (DOC) [file pone.0031813.s013.doc]

| MI-based normalised metric *D* | LM | NNI | DGC |
| --- | --- | --- | --- |
| LM | - | D=0,0019 | D=0,00004 |
| NNI |  | - | D = 0,0154 |
| DGC |  |  | - |

**Supplementary table S3. MI-based normalised *D* measures of the three types of spindle prediction datasets.** The table shows the MI-based normalised metric *D*; D=0 means complete conditional independence, and value of D=1 is the maximum conditional dependence. DGC: GOSS, DORA, CODA, hiPPI and GECO methods. LM: includes CO-CITE method. NNL: Includes MLNN method (see Methods and Supplementary Methods).
