# Supplementary material for: Uncovering the Molecular Machinery of the Human Spindle—An Integration of Wet and Dry Systems Biology
Source: PLoS One. 2012 Mar 9;7(3):e31813. doi: 10.1371/journal.pone.0031813 (PMC3302876; doi:10.1371/journal.pone.0031813)
Supplement: Table S4 — Study of dependencies amongst the individual prediction methods. (DOC) [file pone.0031813.s014.doc]

|  | GOSS | CODApfam | CODAcath | DORA | hiPPI | CO-CITE | MLNN |
| --- | --- | --- | --- | --- | --- | --- | --- |
| GOSS | - | 0,0000001 | 0,0000001 | 0,0000001 | 0,0000001 | 0,0000001 | 0,0000001 |
| CODApfam | - | - | 0,002256 | 0,005719 | 0,0022 | 0,000035 | 0,000677 |
| CODAcath | - | - | - | 0,000095 | 0,00463 | 0,00019 | 0,0005 |
| DORA | - | - | - | - | 0,00000533 | 0,00195 | 0,00226 |
| hiPPI | - | - | - | - | - | 0,00014 | 0,0009 |
| LM | - | - | - | - | - | - | 0,001 |

**Supplementary Table S4. Study of dependencies amongst the individual prediction methods**. The table shows the MI-based normalised metric *D* across all pairs of methods; D=0 means complete conditional independence, and value of D=1 is the maximum conditional dependence. Single methods that were combined into the corresponding high-level predictor given in the main manuscript (see methods section). The D values remains low suggesting a minimal overlap of features. In order to meet the statistical requirements for integrating the predicted datasets it is important to ascertain whether integration improvements could be an artefact caused by correlation and dependencies between the prediction methods.
