# Supplementary material for: Uncovering the Molecular Machinery of the Human Spindle—An Integration of Wet and Dry Systems Biology
Source: PLoS One. 2012 Mar 9;7(3):e31813. doi: 10.1371/journal.pone.0031813 (PMC3302876; doi:10.1371/journal.pone.0031813)
Supplement: Table S7 — Summary of the mitotic phenotype observed upon depletion by siRNA of the selected predicted spindle proteins. (DOCX) [file pone.0031813.s017.docx]

| Protein Name | Uniprot Accession | Uniprot name | Description of mitotic siRNA phenotype |
| --- | --- | --- | --- |
| SHC SH2 domain-binding protein 1 | Q8NEM2 | SHCBP_HUMAN | no obvious phenotype |
| WD repeat protein 76 | Q9H967 | WDR76_HUMAN | slight mitotic delay, broad metaphase plate |
| KIAA0841 | O94927 | K0841_HUMAN | mitotic delay, reduced MTs within the spindle (also [[7](#_ENREF_7)]) |
| GAS2-like protein 3 | Q86XJ1 | GA2L3_HUMAN | slight increase in binucleated cells and nuclear reformation defects (possible cytokinesis defect according to [[7](#_ENREF_7)]) |
| KIAA1794 | Q9NVI1 | K1794_HUMAN | nd |
| Ser/thr-protein phosphatase 1 reg.sub. 10 | Q96QC0 | PP1RA_HUMAN | nd |
| C1orf48 (positive control) | Q96IY1 | CA048_HUMAN | strong mitotic delay, strong congression defects |
| Echinoderm microtubule-associated protein-like 3 | Q32P44 | EMAL3_HUMAN | mitotic delay, chromosome alignment defects [[8](#_ENREF_8)] |
| Pescadillo homologue 1 | O00541 | PESC_HUMAN | faster mitotic progression. Chromosome alignment defects (also shown in yeast in [[9](#_ENREF_9)] |
| Tyrosine kinase p59fyn | P06241 | FYN_HUMAN | faster mitotic progression |
| MORC family CW-type zinc finger protein 2 | Q9Y6X9 | MORC2_HUMAN | mitotic delay, congression defects |
| Nucleoporin 88 | Q99567 | NUP88_HUMAN | mitotic delay, congression defects. |
| Coiled-coil domain containing 99 (hSpindly) | Q96EA4 | CCD99_HUMAN | strong mitotic delay, congression defects [[10](#_ENREF_10)], (also shown in D. melanogaster and C. elegans , [[11](#_ENREF_11),[12](#_ENREF_12)], respectively) |
| Mitogen-activated protein kinase 13 | O15264 | MK13_HUMAN | mitotic delay, chromosome congression defects |
| KIAA1967- p30 DBC protein | Q8N163 | K1967_HUMAN | slight mitotic delay, congression defects |
| WD repeat protein 75 | Q8IWA0 | WDR75_HUMAN | chromosome segregation defects (also shown in D. melanogaster , [[13](#_ENREF_13)] |
| Putative Nucleoporin protein 54 | Q7Z3B4 | NUP54_HUMAN | nd |
| C15orf23 | Q9Y448 | T4AF1_HUMAN | mitotic delay, chromosome congression defects, cytokinesis defects (possibly also according to [[7](#_ENREF_7)]) |
| ZMYM1 protein | Q8N3X8 | Q8N3X8_HUMAN | nd |
| C14orf106 | Q6P0N0 | CV106_HUMAN | severe defects in chromosome alignment [[14](#_ENREF_14)] |

**Supplementary Table S7. Summary of the mitotic phenotype observed upon depletion by siRNA of the selected predicted spindle proteins**
