# Supplementary material for: Uncovering the Molecular Machinery of the Human Spindle—An Integration of Wet and Dry Systems Biology
Source: PLoS One. 2012 Mar 9;7(3):e31813. doi: 10.1371/journal.pone.0031813 (PMC3302876; doi:10.1371/journal.pone.0031813)
Supplement: Table S8 — Specific siRNA oligonucleotides sequences used in this study. (DOCX) [file pone.0031813.s018.docx]

| Protein Name | Target sequence #1 | Target sequence #2 |
| --- | --- | --- |
| SHC SH2 domain-binding protein 1 | CTGGGTGTTCTTGTTATCATA | AAGCAGTTTGTCAAATTGTAA |
| WD repeat protein 76 | AAGGGATTATCTAGCATTAAA | ATGAGGTAAATGAATATAAA |
| KIAA0841* | CAGAGTTTACAAGATAAGGAA | CCAGACCTGCTTTGTATTTAA |
| GAS2-like protein 3 | AACCAAATAAGTGTTCAGGAA | AATAAGAATGCTTCATGGAAA |
| KIAA1794 | nd | nd |
| SerThr-protein phosphatase 1 reg. sub. 10 | nd | nd |
| C1Orf48 (Mis12; positive control) | TAGTTTGATTTATCAAATTAA | AACATTTAGATTACAGCCTAA |
| EML3 | nd | nd |
| Pescadillo homologue 1 | CACCTTGAAGCTGGAGGATAA | CTCGACCACATCATCAAGGAA |
| Tyrosine kinase p59fyn | CCGAGTATATGAACAAAGGAA | AAAGGAAGAGCTCTGAAATTA |
| MORC family CW-type finger protein 2 | ATCGATATCATTATAAGGTTA | AACATTGGTGATCATCTTCAA |
| Nucleoporin 88 | AAGGCTTAAACCCATATTGTA | AAGGGAAATGGTGAAGCAAAT |
| Coiled-coil domain containing 99 | [[10](#_ENREF_10)] | [[10](#_ENREF_10)] |
| Mitogen-activated protein kinase 13 | Catalog number: SI00042672 (Qiagen) | CAGCCGTTTGATGATTCCTTA |
| KIAA1967 | CCCGGTTCCACTTAACAACTA | CCCGCTTATAGTTCGAAGGTA |
| WD repeat protein 75 | CAAGAATATATTAATGATTAT | AACCAAATAAGTGTTCAGGAA |
| Putative Nucleoporin protein 54 | nd | nd |
| C15Orf23 | CCCGGTTCCACTTAACAACTA | CCCGCTTATAGTTCGAAGGTA |
| ZMYM1 protein | nd | nd |
| C14Orf106 | CAAGTAAATAATACTATTCAA | [[14](#_ENREF_14)] |

**Supplementary Table S8. Specific siRNA oligonucleotides sequences used in this study.**
