# Supplementary material for: Uncovering the Molecular Machinery of the Human Spindle—An Integration of Wet and Dry Systems Biology
Source: PLoS One. 2012 Mar 9;7(3):e31813. doi: 10.1371/journal.pone.0031813 (PMC3302876; doi:10.1371/journal.pone.0031813)
Supplement: Table S11 — Results of the Runstest scores run for the all-Mitocheck phenotypes rank. (DOC) [file pone.0031813.s021.doc]

|  | Runstest  Pvalues at sl=0.001 |
| --- | --- |
| Black (full ranked) | 7.4952 10^-3^ |
| Red (subset ranked) | 8.0836 10^-197^ |

**Table S11. Results of the Runstest.** Runstest Pvalues for the all-Mitocheck phenotypes ranked list (first row in the table, and black line in Figure S2) and the Mitocheck spindle-related subset ranked list (second row in the table and red line in Figure S2) at sl= 0,001 significance level to reject H_0_ being true.
