# Supplementary material for: Uncovering the Molecular Machinery of the Human Spindle—An Integration of Wet and Dry Systems Biology
Source: PLoS One. 2012 Mar 9;7(3):e31813. doi: 10.1371/journal.pone.0031813 (PMC3302876; doi:10.1371/journal.pone.0031813)
Supplement: Text S1 — Supporting Materials and Methods. (DOC) [file pone.0031813.s022.doc]

**Supporting documents for “Uncovering the molecular machinery of the human spindle: an integration of wet and dry systems biology”.**

**1) Supplementary Methods:**

**1. The external validation data set (EXPERT dataset).**

The SPIP integrated platform was validated using an external data set. For this purpose, we used a manually generated data set of spindle proteins, the EXPERT dataset (see **Table S10** and **Fig. S5)**.

Generation of this set was guided by results obtained using an automated approach to analyse published articles. This is described below.

As training data we collected 481 articles related to Spindle associated proteins (positive training collection) and 481 articles which were randomly chosen from PubMed and manually examined in order to be sure that they do not contain gene descriptions (this is our negative training collection). The categorisation tool requires a feature definition.

We have used the bag-of-words representation of documents for this setting, which implies that the text of processed articles is decomposed into word tokens (words are used as representational units). The initial set of 12,779 word tokens were then normalized into lower case forms and subjected to stop word filtering using a collection of 696 common stop words (i.e. non-informative words mainly corresponding to function words like articles, pronouns and conjunctions).

We used an additional rule-based filtering step to remove numeric expressions and tokens below a character length cut-off to avoid single letter tokens such as 'a', 'h'. This resulted in a list of 7,645 feature tokens words, out of which 2,782 were finally used by the model of the generated classifier (1,843 positive feature words and 939 negative feature words).

This initial classifier performs with a high recall. The SVM classifier with linear kernel function achieved accuracy on the test set of 95.62% (Precision/recall: 98.93%/92.25%). Nevertheless as the training collection was relatively small, we expected that this classifier would not generalize equally well when applied to the whole PubMed.

The whole PubMed database, containing over 16 million records, was then converted to the corresponding feature vectors and classified using the model derived from the training collection (**Fig. S5**). A total of 525,091 PubMed entries with abstracts have been classified as spindle relevant.

To improve this, by using the concept of spindle child node term mapping (i.e.: selecting the sub-graph of child terms from a given GO spindle term) and additional key words, a second set was generated by reclassifying the whole dataset.

Then, 3,498 articles constitute the training data set, and the whole process is repeated. In the end we extracted 140,000 articles deemed to be relevant for spindle associations. To associate protein records with the spindle-relevant literature, protein normalization using a dictionary lookup approach in combination with a taxonomy-mapping step was used. Preliminary manual inspection of the list was done for the first 30 top ranking proteins.

By manually reviewing the literature, we observed that 26 of them showed a true association to the spindle, displaying relevant characteristics. For instance, one of the top-ranking proteins O75330 (HMMR_HUMAN) shows several associations to spindle in different sentences from different articles. Then, in PubmedId [15498487] “*We have identified the Xenopus ortholog of human RHAMM as a microtubule-associated protein that plays a role in focusing spindle poles and is essential for efficient microtubule nucleation during spindle assembly without centrosomes*”, and this other sentence in PubmedID [15705883] “*RHAMM mitotic localization mirrors that of targeting protein for Xklp2 (TPX2), and RHAMM interacts with the spindle assembly factors dynein and TPX2*.

Further mining of these abstracts, identified 131 genes from 375 sentences in which there is a clear relation to the spindle system. From these 131 proteins, 73 (see **Table S10**) were present in the set of 644 putative spindle proteins identified by Sauer’s proteomics study but had been labelled as unknown i.e. they were missed by Sauer et al. This sub-set of 73 proteins constituted a broad sample of true positive spindle proteins that could be used to benchmark the performance of the integrated methods and to measure the bias (if any) of the proteomics approach. Therefore, these 73 proteins constituted the validation data set (EXPERT), a False Negative (FN) set for Sauer’s experimental assays and a validation data set to estimate the performance of the Fisher integration protocol.

**2. Analysis of the independence of the prediction datasets: Mutual Information measures for pairs of methods.**

The mutual information of two random variables is a quantity that measures the mutual dependence of the two random variables. In a general way, the Mutual Information score expresses the extent to which the observed co-dependence differs from what we would expect by random (statistically speaking). Therefore, the random distribution (H_0_) of the two independent variables is known and implicit in the MI calculation . In our case, we calculate the mutual information using the entropy between the marginal distribution X and its conditional distribution coming from the joint (X,Y) general distribution such as:

I(X;Y)=∑_x∈X_∑_y∈Y_ *p(x,y). p(x,y) / p(x)p(y)* = *H(X)-H(X|Y)* (as *H(X)≥H(X|Y)* then I(X;Y)≥0)

Where H is the entropy and p(x,y) is the joint probability density function of X and Y that can be set to the observed frequencies of protein interaction within the columns of every predictor. i.e., x and y are the p-values associated with the possible scores returned by a predictor.

Hence, we calculated entropies for each pair of methods prediction pvalue datasets, stored in a Matrix MIij, where (ij) corresponds to a pair of positions (i,j). Then we introduced a universal metric d and worked out a normalised MI-based distribution D of pvalues. Our implementation is similar to randomising the MI columns to get a null model, removing any dependency between any two columns i and j .

From this distribution we can easily compute the statistical significance D values (shown in **Tables S3** and **S4**) for the prediction datasets of any pair of methods in the MI matrix.

**3. Searching for novel spindle proteins with the SPIP integrated platform**

The benchmarking of SPIP indicated that SPIP performs well in predicting known spindle proteins having spindle associated phenotypes identified by siRNA interference experiments. However, in order to assess the value of SPIP for predicting novel spindle proteins, we needed to compare the SPIP success rate against that of previous approaches using the same starting dataset of functionally uncharacterised proteins and we needed to subject the selected targets to the same experimental tests. We therefore decided to compare the SPIP performance against the success rate achieved by Sauer et al. in selecting novel spindle targets (by employing expert analysis and simple database searches) from a set of 158 uncharacterised, but putative spindle-associated, proteins identified by their proteomics studies.

We therefore ran SPIP against the same set of 158 proteins (**Table S6**), described as “Unknown function” in the initial set of 765 proteins identified by Sauer et al from their proteomics experiment (SAUER dataset, see **Table S10**) . Sauer and co-workers tested 17 of these proteins of unknown function, selected as likely spindle associated proteins by expert analysis of the corresponding sequences and database annotations. 6 of these 17 proteins were shown to localize to the mitotic spindle apparatus, reflecting a ~35% success rate .

**4. A prediction web server**

The positive results obtained by our integrated predictors encouraged us to generalize the predictions by building an automatic web system able to predict the functional class based on the properties of any seed data set provided by the user. However, it is only possible to integrate methods that perform automatically without intervention (i.e. CODA, hiPPI, GECO, GOSS and COCITE) and those that require careful training and extensive pre-calculation (i.e. LMNN and DORA methods) are unfortunately excluded. Nevertheless the web server provides a valuable alternative predictor of functional associations but it is unlikely to achieve the accuracy of the spindle specific predictor.

The web server allows users to upload a set of query proteins, and specify a reference set for a biological system of interest. The reference set may be specified by providing a Gene Ontology term (the server will gather proteins annotated with this term) or by manually providing a list of proteins. Integrated predictions will then rank the query proteins by the strength of their association with the reference set. An option to view the results as an interactive graphical network diagram is provided.

Additionally, we provide pre-computed spindle predictions using the complete set of methods and the integrated predictor described in this paper, including the computationally expensive methods LMNN and DORA. In this instance, users need only to upload a set of query proteins; these are then ranked by their probability of being spindle proteins. In both modes (general-purpose and spindle-only) the query proteins can come from anywhere in the human proteome. The server, its technical details and user instructions are available at http://predster.cathdb.info/, and will be described in a technical publication in due time.

**5. Using Mitocheck Data to Validate the Performance of SPIP in the Whole Human Proteome.**

All the experimentally assayed genes in the Mitocheck experiment , with an associated phenotype, were mapped onto the SPIP ranked list of spindle predictions for the whole human proteome (human spindle ranked list). Those genes related to more than one protein product in the ranked list were not considered due to the difficulty in making unambiguous assignments. In total 1,660 mitocheck genes, with associated phenotypes, were mapped onto the list of 32,145 ranked human proteome spindle predictions.

Since Mitocheck also contains phenotype categories which are not specific to spindle genes, such as “Cell death” or “Large”, we mapped the human spindle ranked list with a subset of Mitocheck phenotype categories more closely related to spindle gene malfunctions in the cell cycle, such as: “Segregation problems”, “Metaphase alignment problems” and “Metaphase delay/arrest”. In total 361 mitocheck genes with any of these phenotypes were mapped onto the human proteome spindle ranked list.

We compared the distributions of all the Mitocheck phenotype matches and the subset of Mitocheck spindle related phenotype matches (True Positive -TP- hits) with a Random distribution (used to calculate the False Positives -FP- hits) in the human spindle ranked list. Enrichment was calculated by dividing the number of TPs by the number of FPs found at the same rank threshold (see **Fig. S2**). For example, an enrichment score of 9 associated with a rank threshold ≤ 89 means we find 9 times more TPs in the set of predictions with rank ≤ 89 than a random predictor does by chance.

These results demonstrate that enrichment of siRNA experiments, associated with the Mitocheck spindle related phenotypes, is significantly higher at the top of the ranked list (enrichment value about 10; see red line in **Fig. S2**) compared to all Mitocheck phenotypes (enrichment value about 3; see black line in **Fig. S2**). This suggests that by applying the SPIP integrated spindle predictor to select targets one could expect a significant improvement in identifying siRNA validation experiments with positive spindle-related phenotypes.

In order to demonstrate the statistical significance of the Mitocheck enrichment analysis two hypothesis tests were undertaken: the Runstest, which measures the statistical significance of the TP distribution pattern throughout the ranked list; and the Random test, which estimates the random probability of obtaining TP/FP enrichment ratios equal to or greater than those observed.

The Runstest statistical assessment tests whether the distribution patterns of two given variables (e.g. variable 1: Rank positions with TPs, and variable 2: rank positions without TPs) are in a random order in the ranked list against the alternative that the ordering is not random. In Statistics this is a test that takes values in a random variable X, assuming that they come in a random order as the null hypothesis, against the alternative that they do not. The test is based on the number of runs of consecutive values above or below the mean of X. Then one checks whether the size of the resulting set is smaller or larger than expected by random.

The Runstest analysis demonstrates that the TPs rank distributions for both the ‘all set’ and the ‘spindle-related-subset’ of the Mitocheck data are not due to random organization (see Runstest Pvalues at sl=0.001 in Table S11). The Runstest Pvalue score for the Mitocheck spindle-subset is about 10^194^ magnitudes smaller than the all Mitocheck Runstest Pvalue, indicating that SPIP seems to perform significantly better when assessing the prediction with this more specific spindle-related subset of Mitocheck experiments.

The Runstest analysis proves the non-random character of the distribution of TPs but not the trend of this pattern in the ranked list (e.g. top, bottom etc.). The Random-test provides us with the trend of the non-random distribution pattern identified by the Runtest.

The Random-test generated 10,000 random iterations of the cumulative TPs distribution for each of the two Mitocheck ranked lists (all Mitocheck: black and spindle related subset: red in Figure S2). Based on these 10,000 random distributions we calculated the random probability of retrieving TP/FP enrichment ratios equal to or greater than those observed in each of the two Mitocheck ranks. The Random-test indicates a significant non-random enrichment of TPs at the top of both ranked lists, this enrichment being much more significant in the spindle-related subset than for the full Mitocheck set as expected. (**Figure S9**).

The combination of these two tests (Runtest and Random test) demonstrate the significant enrichment of TPs (Mitocheck phenotypes) at the top of the two Mitocheck enrichment validation ranked lists, with this enrichment being significantly higher for the validation performed with the spindle related subset from Mitocheck.

**6. Mapping Mitocheck data onto the SPIP ranked list of 158 unknown proteins from the Sauer study.**

All the genes experimentally assayed in the Mitocheck experiment were also mapped onto the Sauer set of 158 unknown proteins identified by a proteomics experiment (SPIP158, **Table S6**). This mapping showed that 84% of the genes in the 158 dataset had been tested by the Mitocheck experiments (**Table S6**). However, many of the Sauer set genes did not yield a phenotype when inhibited by siRNA in the Mitocheck experiment.

We divided the SPIP ranked list into three equivalent stretches (from rank 1 to 52, from 53 to 105, and from 106 to 158) and compared the distribution of all the Mitocheck phenotypes (21 genes in total) and the subset of Mitocheck spindle related phenotypes (6 in total). We observed that in all cases the distribution of Mitocheck gene phenotypes was much higher at the top of the list than in the lower ranges (**Fig. S3**). These results complement our experimental validation, giving additional support for the good performance of the SPIP platform in predicting new experimental targets involved in cell cycle and spindle.

**7. Calculation and ranking of the spindle hidden hubs**

To predict hidden spindle hubs we took the ‘top 2%’ of proteins (642 proteins) from the SPIPall ranked list in the human proteome, as representative of proteins highly likely to be spindle associated. We selected the top 2% in order to apply a highly restrictive threshold for obtaining the most significant SPIP predictions, with which to look further for spindle hidden hubs.

We constructed a spindle sub-network by combining the 149 known spindle proteins from the Sauer set (SEED) with the list of 73 EXPERT proteins (total of 223 curated spindle proteins). The 73 proteins from the EXPERT dataset present in the top-2% of the SPIPall dataset were removed to avoid redundancy. We then assembled two independent protein-protein interaction networks in the human proteome (1) from a combination of all experimental datasets - ‘Knowledgegram’ (KG) and (2) from a combination of all predicted datasets - Predictogram (PG).

We did not use, for example, the MLNN or DORA methods to generate the Predictogram, since they give probabilities of being spindle associated for individual targets but do not provide predictions about possible interacting partners. The text mining predictions were also not included since these methods mine knowledge in literature and cannot be considered as pure *ab-initio*.

After removing the EXPERT proteins from the top-2% set and also those proteins without interaction partners in the *ab-initio* prediction set (i.e. GECO, hiPPI, CODAcath and CODApfam), the top-2% list of proteins is reduced from 642 to 530 proteins.

We calculated the number of different connections (ki) (in both the KG and the PG) between the 223 bait proteins and the 530 top-2% and between the bait proteins and the human proteome.

We then identified and ranked ‘hidden’ spindle hubs as those targets with low ki in the KG dataset (KG_ki) but high ki in the PG (PG_ki) and with a high percentage of the ki connections specific to spindle partners i.e the 223 known spindle sub-network set (% Spindle_specific). We applied a simple prioritisation criteria for ranking these targets: 1) Selecting targets with KG_ki<=10 and PG_ki>=50, and then ranking them by their % Spindle_specific values (see **Table S9**). Functional analysis of the hidden spindle hubs was performed using literature information, while significant keywords, clustering of functional classes and enrichment of the spindle hidden hubs was performed by using the DAVID Server, a highly cited and widely used resource for functional analysis of protein sets .

**8. Non-hub hidden spindle proteins analysis.**

We wanted to check whether the network analysis is valuable or superfluous for predicting spindle hidden hubs in the top 2% of the SPIP ranked. In order to answer this question we looked for “non-hub hidden spindle” proteins, defining “non-hub hidden spindle” proteins as those proteins belonging to the same top 2% of the ranked list which show no interactions in the KG network (i.e. hidden proteins) and just 1 predicted interaction in the PG network (not hubs).

We found 19 of these “non-hub hidden spindle hidden” proteins and labelled their position in the top of the 2% ranked list, doing the same thing for the 6 “hidden spindle hubs” studied in this work (see Figure S10). From the rank distributions of both sets it is clear that hub proteins are not always higher in the ranked list than non-hubs and without the network analysis it is not possible to recognise these hidden spindle hubs in the top 2% of the SPIP ranked list.

**9. Assessing the performance of COCITE method.**

In the CoCITE gene network, we recovered 147 of the 149 SEED proteins labelled as True Spindle in the Sauer et al. work . To estimate the ability of our scoring system to capture true spindle proteins, we considered each “SEED” protein as a “non-SEED” protein and calculated the scores for it. We ranked all the human proteins in our network according to the calculated scores S1, S2 and the combination of both scores (St=S1+S2). We performed a ROC analysis for each of the ranked lists by considering SEED proteins as positive cases and non-SEED proteins as negative ones. We observed that all three scores (S1, S2, St) were able to discriminate positive from negative cases (see **Fig. S8**). Interestingly, we observed that S2 (AUC=0.83) outperformed S1 (AUC=0.76). This difference is mainly explained by the higher sensitivity of the S2 score (see **Fig. S8**). Even more relevant is that St outperforms (AUC≤0.97) both S1 and S2, showing an impressive capability of recovering positive cases. It is important to consider that an important part of the success of these approaches comes from the high quality of the COCITE network and from the fact that spindle proteins seem to be well represented in our network.

Finally, we used St for all the human proteins in our network in order to establish whether their association to the True Spindle set (SEED set) is supported by the COCITE network.

**Supplementary methods references**

1. Sauer G, Korner R, Hanisch A, Ries A, Nigg EA, et al. (2005) Proteome analysis of the human mitotic spindle. Mol Cell Proteomics 4: 35-43.

2. Brillinger DR (2004) Some data analyses using mutual information. Brazilian Journal of Probability and Statistics 18: 163–182.

3. Hamacher K (2008) Relating sequence evolution of HIV1-protease to its underlying molecular mechanics. Gene 422: 30-36.

4. Neumann B, Walter T, Heriche JK, Bulkescher J, Erfle H, et al. (2010) Phenotypic profiling of the human genome by time-lapse microscopy reveals cell division genes. Nature 464: 721-727.

5. Siegel S (1957) Nonparametric Statistics. The American Statistician 11: 13-19.

6. Hwang D, Rust AG, Ramsey S, Smith JJ, Leslie DM, et al. (2005) A data integration methodology for systems biology. Proc Natl Acad Sci U S A 102: 17296-17301.

7. Kittler R, Pelletier L, Heninger AK, Slabicki M, Theis M, et al. (2007) Genome-scale RNAi profiling of cell division in human tissue culture cells. Nat Cell Biol 9: 1401-1412.

8. Tegha-Dunghu J, Neumann B, Reber S, Krause R, Erfle H, et al. (2008) EML3 is a nuclear microtubule-binding protein required for the correct alignment of chromosomes in metaphase. J Cell Sci 121: 1718-1726.

9. Killian A, Le Meur N, Sesboue R, Bourguignon J, Bougeard G, et al. (2004) Inactivation of the RRB1-Pescadillo pathway involved in ribosome biogenesis induces chromosomal instability. Oncogene 23: 8597-8602.

10. Chan YW, Fava LL, Uldschmid A, Schmitz MH, Gerlich DW, et al. (2009) Mitotic control of kinetochore-associated dynein and spindle orientation by human Spindly. J Cell Biol 185: 859-874.

11. Schmidt JC, Kiyomitsu T, Hori T, Backer CB, Fukagawa T, et al. (2010) Aurora B kinase controls the targeting of the Astrin-SKAP complex to bioriented kinetochores. The Journal of cell biology 191: 269-280.

12. Griffis ER, Stuurman N, Vale RD (2007) Spindly, a novel protein essential for silencing the spindle assembly checkpoint, recruits dynein to the kinetochore. J Cell Biol 177: 1005-1015.

13. Somma MP, Ceprani F, Bucciarelli E, Naim V, De Arcangelis V, et al. (2008) Identification of Drosophila mitotic genes by combining co-expression analysis and RNA interference. PLoS genetics 4: e1000126.

14. Fujita Y, Hayashi T, Kiyomitsu T, Toyoda Y, Kokubu A, et al. (2007) Priming of centromere for CENP-A recruitment by human hMis18alpha, hMis18beta, and M18BP1. Dev Cell 12: 17-30.
